# Hydroxychloroquine with or without azithromycin for treatment of early SARS-CoV-2 infection among high-risk outpatient adults: A randomized clinical trial

**DOI:** 10.1016/j.eclinm.2021.100773

**Published:** 2021-02-27

**Authors:** Christine Johnston, Elizabeth R. Brown, Jenell Stewart, Helen C.Stankiewicz Karita, Patricia J. Kissinger, John Dwyer, Sybil Hosek, Temitope Oyedele, Michael K. Paasche-Orlow, Kristopher Paolino, Kate B. Heller, Hannah Leingang, Harald S. Haugen, Tracy Q. Dong, Anna Bershteyn, Arun R. Sridhar, Jeanne Poole, Peter A. Noseworthy, Michael J. Ackerman, Susan Morrison, Alexander L. Greninger, Meei-Li Huang, Keith R. Jerome, Mark H. Wener, Anna Wald, Joshua T. Schiffer, Connie Celum, Helen Y. Chu, Ruanne V. Barnabas, Jared M. Baeten, for the COVID-19 Early Treatment Study Team

**Affiliations:** a Division of Allergy and Infectious Diseases, University of Washington, United States; b Department of Laboratory Medicine and Pathology, University of Washington, United States; c Department of Biostatistics, University of Washington, United States; d Department of Epidemiology, University of Washington, United States; e Department of Global Health, University of Washington, United States; f Division of Cardiology, University of Washington, United States; g Division of Rheumatology, University of Washington, Seattle, WA, United States; h Vaccine and Infectious Disease Division, Fred Hutchinson Cancer Research Center, Seattle, WA; i Public Health Sciences Division, Fred Hutchinson Cancer Research Center, Seattle, WA, United States; j School of Public Health and Tropical Medicine, Tulane University, New Orleans, LA, United States; k School of Medicine, Tulane University, New Orleans, LA, United States; l John H. Stroger, Jr., Hospital of Cook County, Chicago, IL, United States; m Rush University Medical Center, Chicago, IL, United States; n Boston University School of Medicine, Boston, MA, United States; o Boston Medical Center, Boston, MA, United States; p State University of New York Upstate Medical University, Syracuse, NY, United States; q New York University Grossman School of Medicine, NY, NY, United States; r Mayo Clinic, Rochester, MN, United States

**Keywords:** Coronavirus, COVID-19, SARS-CoV-2, Hydroxychloroquine, Azithromycin, Early treatment, Remote enrollment, Randomized controlled trial

## Abstract

**Background:**

Treatment options for outpatients with COVID-19 could reduce morbidity and prevent SARS-CoV-2 transmission.

**Methods:**

In this randomized, double-blind, three-arm (1:1:1) placebo-equivalent controlled trial conducted remotely throughout the United States, adult outpatients with laboratory-confirmed SARS-CoV-2 infection were recruited. Participants were randomly assigned to receive hydroxychloroquine (HCQ) (400 mg BID x1day, followed by 200 mg BID x9days) with or without azithromycin (AZ) (500 mg, then 250 mg daily x4days) or placebo-equivalent (ascorbic acid (HCQ) and folic acid (AZ)), stratified by risk for progression to severe COVID-19 (high-risk vs. low-risk). Self-collected nasal swabs for SARS-CoV-2 PCR, FLUPro symptom surveys, EKGs and vital signs were collected daily. Primary endpoints were: (a) 14-day progression to lower respiratory tract infection (LRTI), 28-day COVID-19 related hospitalization, or death; (b) 14-day time to viral clearance; secondary endpoints included time to symptom resolution (ClinicalTrials.gov: NCT04354428). Due to the low rate of clinical outcomes, the study was terminated for operational futility.

**Findings:**

Between 15th April and 27th July 2020, 231 participants were enrolled and 219 initiated medication a median of 5.9 days after symptom onset. Among 129 high-risk participants, incident LRTI occurred in six (4.7%) participants (two control, four HCQ/AZ) and COVID-19 related hospitalization in seven (5.4%) (four control, one HCQ, two HCQ/AZ); no LRTI and two (2%) hospitalizations occurred in the 102 low-risk participants (one HCQ, one HCQ/AZ). There were no deaths. Among 152 participants with viral shedding at enrollment, median time to clearance was 5 days (95% CI=4–6) in HCQ, 6 days (95% CI=4–8) in HCQ/AZ, and 8 days (95% CI=6–10) in control. Viral clearance was faster in HCQ (HR=1.62, 95% CI=1.01–2.60, *p* = 0.047) but not HCQ/AZ (HR=1.25, *p* = 0.39) compared to control. Among 197 participants who met the COVID-19 definition at enrollment, time to symptom resolution did not differ by group (HCQ: HR=1.02, 95% CI-0.63–1.64, *p* = 0.95, HCQ/AZ: HR=0.91, 95% CI=0.57–1.45, *p* = 0.70).

**Interpretation:**

Neither HCQ nor HCQ/AZ shortened the clinical course of outpatients with COVID-19, and HCQ, but not HCQ/AZ, had only a modest effect on SARS-CoV-2 viral shedding. HCQ and HCQ/AZ are not effective therapies for outpatient treatment of SARV-CoV-2 infection.

**Funding:**

The COVID-19 Early Treatment Study was funded by the Bill & Melinda Gates Foundation (INV-017062) through the COVID-19 Therapeutics Accelerator. University of Washington Institute of Translational Health Science (ITHS) grant support (UL1 TR002319), KL2 TR002317, and TL1 TR002318 from NCATS/NIH funded REDCap. The content is solely the responsibility of the authors and does not necessarily represent the views, decisions, or policies of the institutions with which they are affiliated. PAN and MJA were supported by the Mayo Clinic Windland Smith Rice Comprehensive Sudden Cardiac Death Program.

**Trial registration** ClinicalTrials.gov number NCT04354428

**Research in context**

**Evidence before this study**

The severe acute respiratory syndrome coronavirus 2 (SARS-CoV-2) causes coronavirus-19 (COVID-19). Rapid global spread of SARS-CoV-2 resulted in a pandemic that has taken nearly 2 million lives and altered all aspects of daily life during 2020. Repurposed drugs to prevent disease progression and mortality were sought immediately to ameliorate the course of infection, particularly among people with early or mild disease in the non-hospitalized setting. Hydroxychloroquine (HCQ) with or without azithromycin (AZ) were used empirically given in vitro data that they could inhibit SARS-CoV-2. A PubMed literature search on 14 Jan 2021 with the search terms COVID-19 OR SARS-CoV-2 AND hydroxychloroquine revealed 2144 results; when limited to randomized controlled trials, only 30 results remained. Of these, three trials treated people with mild-moderate disease with HCQ or HCQ/AZ in the outpatient setting. Studies by Skipper et al. in the United States and Mitjà et al. in Spain studied HCQ in blinded and open-label trials, respectively. Neither study showed any difference in time to symptom resolution; Mitjà et al. also studied SARS-CoV-2 viral loads from nasopharyngeal swabs and showed no difference in quantity of virus detected at days 3 and 7 after randomization. Omrani et al. found that HCQ with or without AZ was not associated with virologic cure at day 6 among non-hospitalized patients.

**Added value of this study**

This RCT of HCQ with or without azithromycin extends prior findings by 1) providing further evidence that HCQ does not affect the clinical course of SARS-CoV-2 in outpatients, 2) providing further evidence that HCQ with azithromycin does not affect SARS-CoV-2 clinical or virologic clearance 3) enriching the study population for a high-risk cohort, which would be more likely to demonstrate a clinical benefit if one existed, and 4) providing robust virologic data with daily self-collected nasal swabs.

**Implications of all the available evidence**

HCQ and HCQ with azithromycin do not affect the clinical course of COVID-19 among outpatients and should not be used to treat SARS-CoV-2 infection.

Alt-text: Unlabelled box

## 1 Introduction

Severe Acute Respiratory Syndrome Coronavirus 2 (SARS-CoV-2), which causes COVID-19, has spread rapidly throughout the world since December 2019 [1,2]. Steroids and remdesivir have benefit for hospitalized patients, but no effective outpatient treatments have been established [3,4]. SARS-CoV-2 transmission, infection, and recovery mostly occurs in outpatient settings; over 80% of cases are mild[5]. Therapies initiated in early infection that decrease the risk of hospitalization or chronic symptoms and minimize onward transmission are needed, particularly for individuals with risk factors for severe COVID-19 [6].

Hydroxychloroquine (HCQ) has in vitro activity against SARS-CoV-2 [7], and HCQ with azithromycin (AZ) was hypothesized to shorten the duration of viral shedding based on observational studies [8], [9], [10], [11]. Three randomized trials of HCQ treatment of COVID-19 in outpatients have been performed in predominantly younger populations without co-morbidities associated with severe COVID-19. These trials showed that HCQ did not decrease the duration of COVID-19 symptoms [12,13], or decrease the quantity of SARS-CoV-2 detected in nasal swabs [13] and a third trial of HCQ with or without AZ did not show a significant difference in proportion of participants with viral clearance at study day 6 [14]. We hypothesized that studying HCQ with or without AZ in an outpatient population enriched for those at high-risk for progression to COVID-19 would provide additional information about the utility of these interventions to prevent progression to lower respiratory tract infection (LRTI), hospitalization, or death. We evaluated the efficacy of HCQ and HCQ+AZ to prevent progression of COVID-19 and decrease time to SARS-CoV-2 clearance from nasal swabs among high- and low-risk outpatients with laboratory-documented SARS-CoV-2 infection.

## 2 Methods

### 2.1 Study design

The study was a randomized, double-blind 3-arm trial, with 1:1:1 randomization to HCQ + folic acid (FA), HCQ and AZ, and ascorbic acid (AA) and FA as a placebo-equivalent control. Five institutions in the United States conducted the study with a remote protocol (Supplement). Study visits were conducted via Health Insurance Portability and Accountability Act (HIPAA)-compliant telemedicine. The Western Institutional Review Board (WIRB) approved this study with reliance agreements with collaborating institutions. The study was registered at ClinicalTrials.gov (NCT04354428).

### 2.2 Participants

Participants were recruited through social media or at local sites. Eligibility criteria included age between 18 and 80 years old, laboratory-confirmed SARS-CoV-2 infection diagnosis within the prior 72 h, able to provide informed consent in English or Spanish and to participate in telehealth visits. Pregnant and lactating persons were eligible. The high-risk cohort enrolled participants with established risk factors for severe COVID-19 including age 60 years or greater; pulmonary disease; diabetes mellitus, hypertension, or self-reported body mass index ≥30 kg/m^2^. Persons who did not meet any of these criteria were enrolled into the low-risk cohort. Exclusion criteria included cirrhosis, stage IV kidney disease, coronary artery disease, and certain medications that were associated with torsades de pointes or interacted with study medications (Detailed in Protocol Section 7.9.1). After counseling about the intervention, study procedures, and risks, electronic, written informed consent was obtained from all participants.

### 2.3 Randomization and masking

Study medications were dispensed according to a computer-generated randomization sequence. To prevent unblinding, randomization occurred by household 1:1:1 to the following interventions, stratified by site and high- or low-risk cohort: 1) HCQ+FA, 2) HCQ+AZ, or 3) AA+FA. AA served as the placebo-equivalent for HCQ, with similar color and both with distinctive taste, and FA was the placebo-equivalent for AZ. The oral dosing regimen was as follows: HCQ 400 mg (or AA 500 mg) twice on Day 1, followed by HCQ 200 mg (or AA 250 mg) twice daily for 9 days plus AZ 500 mg (or FA 800 µg) once on Day 1, followed by AZ 250 mg (or FA 400 µg) once daily for 4 days.  The first participant enrolled per household defined the randomization of other household members.

### 2.4 Procedures

After enrollment, a courier delivered study medication, instructions, nasal swabs and tubes, a pulse oximeter (Vive Precision), KardiaMobile 6-lead Electrocardiogram (EKG) monitor (AliveCor, Mountain View, CA), and digital oral thermometer (Adtemp IV, Hauppauge, NY). Mid-turbinate nasal swabs were self-collected as previously described [15]. Each morning (Days 2–14, 21, and 28) participants received individualized links to surveys to record SpO2, pulse, respiratory rate, temperature, time of swab collection, medication adherence, and symptoms (modified FLUPro) [16]. Evening surveys (Days 2–14) captured vital signs and medication adherence. Study staff were available 24 h/day. Scheduled contact with study clinicians took place on Days 2, 4, 9, 14, and 28 to assess symptoms, medication adherence, and adverse events (AEs). Participants received instructions for returning swabs. Participants completed an Exit Survey at study completion (Day 28). Data were managed using REDCap electronic data capture tools hosted at University of Washington [17,18].

### 2.5 Laboratory methods

Reverse transcription polymerase chain reaction (RT-PCR) testing targeted the SARS-CoV-2 nucleocapsid genes N1 and N2 [19]. RT-PCR was performed on an ABI 7500 real-time PCR system (Applied Biosystems). An internal control amplification, either RNase P or EXO (RNA spike-in), was performed to monitor RNA extraction and RT-PCR quality. Specimens were considered positive if either or both the N1 and N2 targets were detected and the cycle threshold (Ct), a semiquantitative measure of viral load, was ≤40.

### 2.6 Outcomes and assessment

#### 2.6.1 Primary outcomes

The primary clinical outcome was development of LRTI, defined by SpO2<93% on two readings ≥two hours but ≤48 h apart with simultaneous indication of “trouble breathing”, “wet cough” or “dry cough” graded at least “somewhat” on the FLU-Pro-survey through Day 14, COVID-19-related hospitalization, or death. The primary virologic outcome was time to cessation of viral shedding, defined by two consecutive nasal swabs without SARS-CoV-2 detection, through Day 14. Participants were excluded from viral shedding analyses if the first two collected swabs were negative for SARS-CoV-2 or ≤2 swabs were collected.

#### 2.6.2 Secondary outcome

To determine if HCQ or HCQ/AZ was associated with faster symptom resolution among those that had COVID-19 symptoms at baseline, we analyzed the time to COVID-19 symptom resolution by Day 14. COVID-19 was defined as two of the following: fever (≥38 °C), chills, rigors, myalgia, headache, sore throat, new olfactory or taste disorders, or one of the following: cough, shortness of breath or difficulty breathing [20]. COVID-19 symptoms were graded by the highest self-reported symptom on a 5-point scale. Participants who did not meet the definition at screening were excluded from the symptom resolution analysis.

#### 2.6.3 Safety measures

Reportable adverse events (AEs) included serious AEs (SAE), AEs resulting in study medication discontinuation, and AEs assessed related to study medication. QTc was monitored daily via six-lead EKGs. The digital recordings were de-identified and transferred to a central facility (Mayo Clinic, Rochester, MN). The QT intervals were measured by Certified Rhythm Analysis Technicians using Bazett's formula. Readings were returned to the investigator within one hour of receipt. If QTc was >500 ms or >60 ms above the baseline reading, study medication was temporarily withheld and the EKG was repeated. If the QTc abnormality remained, study medication was discontinued.

#### 2.6.4 Statistical analysis

The sample size was estimated based on 30% clinical event rate for the primary clinical outcomes. To detect 50% treatment efficacy with 90% power and two-sided alpha of 0.05, we planned to enroll 165 high-risk participants per randomized group, to provide 93 events per pairwise comparison. In addition, 45 low-risk participants per group were planned for complementary assessment of the viral shedding outcome. Clinical outcomes were summarized by counts and frequency of occurrence. On 27th July 2020, the DSMB reviewed the small number of clinical endpoints in the high-risk cohort and determined that operational futility for the primary clinical outcomes was reached due to low frequency of endpoints. With the low event rate in the high-risk cohort, thousands of participants per arm would need to be recruited to detect a significant difference between the groups, and the decision was made to terminate the trial.

Baseline characteristics were compared across arms using generalized estimating equations with an identity link for continuous characteristics, a log link for count data and a logit link for binary characteristics with exchangeable correlation within households. We used a Cox proportional hazards model to estimate the adjusted hazard ratio of viral clearance or COVID-19 disease resolution at Day 14 in the intervention versus control groups. Both endpoints included pre-specified adjustments for age and sex and stratification by baseline symptom duration (>4 days). Viral clearance was stratified by site to control for delays in return of test results, while disease resolution was stratified by risk cohort. Endpoint time was set at the day of the endpoint-defining event. Participants who did not reach the efficacy endpoints were censored on the last day when endpoint data were ascertained. Corresponding 95% confidence intervals (CI) and Wald test statistics were calculated using robust standard errors from the sandwich estimator to account for correlation from multiple participants within a household [21]. Cumulative incidence and median event time were calculated using the Kaplan-Meier method*.* Low levels of viral shedding may not represent viable virus leading to onward transmission; therefore we conducted a post-hoc sensitivity analysis using Ct<34 as a cut-off for positivity [22]. In addition, we performed a post-hoc sensitivity analysis adjusting for baseline viral load, an approach used in other SARS-CoV-2 clinical trials which use virologic primary outcomes [23]. For all analyses, two-sided p-values<0.05 were considered significant.

Safety analyses included counts of AEs; those related to EKG monitoring were tabulated separately from other AEs.

*Role of the funding source:* The sponsors provided input into study design. The sponsors had no role in data collection, analysis, interpretation, or writing of the report.

## 3 Results

### 3.1 Participants

**Fig. 1:**
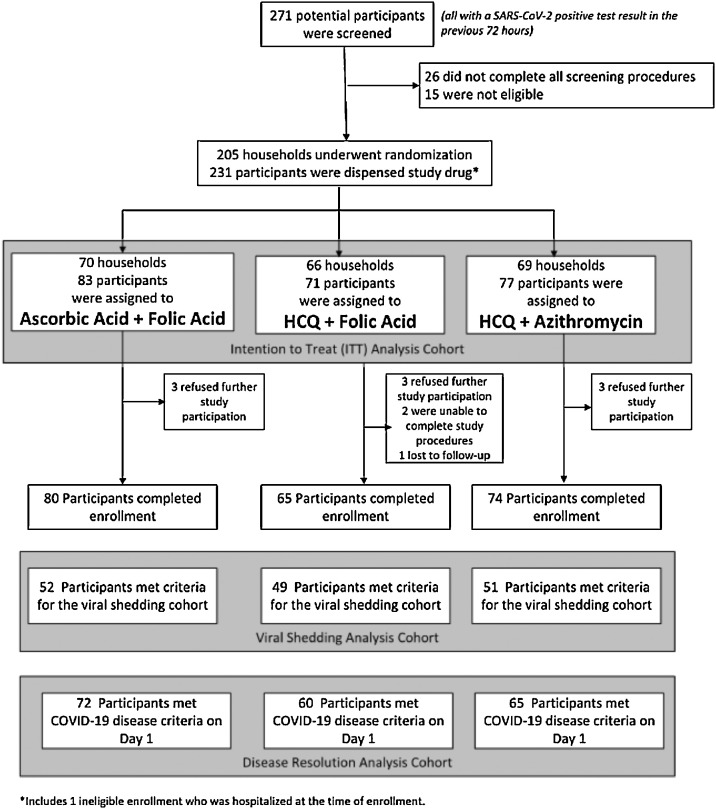
Consort diagram showing enrollment and follow up of participants.

Between 15th April and 27th July 2020, 271 participants were screened and 231 participants from 205 households underwent randomization (Fig. 1). The median age was 37 years (range 18–78) and 131 (56.7%) were women (Table 1). Overall, 117 (50.6%) self-identified as white, 26 (11.3%) as Black, 39 (16.9%) as American Indian/Alaska Native, 11 (4.8%) as Asian, 3 (1.3%) as Native Hawaiian/Pacific Islander, and 32 (13.9%) as “other”; 71 (30.7%) identified as Hispanic/Latinx. The median time between symptom onset and receipt of study medication was 5.9 days (IQR 4.0–8.2). The groups were well balanced at baseline (Table 1, Table S1, S2). One-hundred twenty-nine participants (55.8%) were enrolled into the high-risk cohort (Table 1); 23 (17.8%) were ≥60 years, 98 (76%) had BMI≥30 kg/m^2^, 27 (20.9%) had a hypertension, 17 (13.2%) had diabetes mellitus; and 39 (30.2%) had more than one risk factor (Table S3). The remaining 102 participants were enrolled into the low-risk cohort. Twelve participants (5.2%) refused study participation, were unable to complete study procedures, or were lost to follow up prior to completing the enrollment survey (Fig. 1). Participants reported taking 3515 of 4389 (80.1%) expected medication doses, and adherence rates did not differ by randomized group. The Day 28 survey was completed by 194 participants (84.0%) and 208 (90%) completed the Exit Survey, indicating high participant retention.

**Table 1:** Baseline characteristics of randomized participants by arm.

		**Randomized Arm**				**p-value**⁎⁎	
		**Ascorbic acid+ folic acid**	**HCQ + folic acid**	**HCQ + azithromycin**	**All**		
**Characteristic**	**Category**						
Enrolled (n)	Total	83	71	77	231		
*High Risk Cohort*		*48*	*37*	*44*	*129*		
*Low Risk Cohort*		*35*	*34*	*33*	*102*		
Age (years)	18–29	21 (25.3%)	19 (26.8%)	24 (31.2%)	64 (27.7%)		
	30–39	26 (31.3%)	28 (39.4%)	21 (27.3%)	75 (32.5%)		
	40–49	16 (19.3%)	13 (18.3%)	14 (18.2%)	43 (18.6%)		
	50–59	12 (14.5%)	5 (7.0%)	9 (11.7%)	26 (11.3%)		
	60–69	7 (8.4%)	5 (7.0%)	8 (10.4%)	20 (8.7%)		
	70–80	1 (1.2%)	1 (1.4%)	1 (1.3%)	3 (1.3%)		
	Median	38	36	37	37		0.5997
	Min, max	(18, 70)	(19, 78)	(18, 71)	(18, 78)		
Sex	Born Female	45 (54.2%)	39 (54.9%)	47 (61.0%)	131 (56.7%)		0.6109
	Born Male	38 (45.8%)	32 (45.1%)	30 (39.0%)	100 (43.3%)		
Race	American Indian or Alaska Native	12 (14.5%)	11 (15.5%)	16 (20.8%)	39 (16.9%)		
	Asian	4 (4.8%)	3 (4.2%)	4 (5.2%)	11 (4.8%)		
	Native Hawaiian or other Pacific Islander	2 (2.4%)	0 (0.0%)	1 (1.3%)	3 (1.3%)		
	Black or African American	7 (8.4%)	10 (14.1%)	9 (11.7%)	26 (11.3%)		
	White	41 (49.4%)	37 (52.1%)	39 (50.6%)	117 (50.6%)		
	Other	15 (18.1%)	10 (14.1%)	7 (9.1%)	32 (13.9%)		
	Prefer not to say	2 (2.4%)	0 (0.0%)	1 (1.3%)	3 (1.3%)		
Hispanic or Latina/Latino/Latinx	No	52 (62.7%)	48 (67.6%)	59 (76.6%)	159 (68.8%)		0.2043
	Yes	30 (36.1%)	23 (32.4%)	18 (23.4%)	71 (30.7%)		
	Prefer not to say	1 (1.2%)	0 (0.0%)	0 (0.0%)	1 (0.4%)		
Preferred Language	English	72 (86.7%)	68 (95.8%)	70 (90.9%)	210 (90.9%)		0.1312
	Spanish	11 (13.3%)	3 (4.2%)	7 (9.1%)	21 (9.1%)		
BMI (kg/m2)	<30	48 (57.8%)	43 (60.6%)	42 (54.5%)	133 (57.6%)		0.6887
	≥30	35 (42.2%)	28 (39.4%)	35 (45.5%)	98 (42.4%)		
Symptomatic COVID-19 at screening*	No	6 (7.2%)	5 (7.0%)	8 (10.4%)	19 (8.2%)		0.7218
	Yes	77 (92.8%)	66 (93.0%)	69 (89.6%)	212 (91.8%)		
Time since symptom onset (days)*	Median	5.9	5.9	5.8	5.9		0.5427
	IQR	(4.0, 8.3)	(4.0, 7.8)	(3.9, 8.3)	(4.0, 8.2)		
Completed enrollment	No	3 (3.6%)	6 (8.5%)	3 (3.9%)	12 (5.2%)		0.4439
	Yes	80 (96.4%)	65 (91.5%)	74 (96.1%)	219 (94.8%)		
Hours between screening visit and enrollment survey completion	<24	45 (64.3%)	33 (50.0%)	44 (63.8%)	122 (59.5%)		0.5548
	24 - <48	23 (32.9%)	27 (40.9%)	24 (34.8%)	74 (36.1%)		
	≥48	12 (17.1%)	5 (7.6%)	6 (8.7%)	23 (11.2%)		
							
Households	Total	70	66	69	205		
Number of participants per household	1	60 (85.7%)	61 (92.4%)	61 (88.4%)	182 (88.8%)		
	2	9 (12.9%)	5 (7.6%)	8 (11.6%)	22 (10.7%)		
	3+	1 (1.4%)	0 (0.0%)	0 (0.0%)	1 (0.5%)		

⁎ Criteria for symptomatic COVID-19 met at screening. Time since symptom onset computed as the difference in hours between date and time of (any) symptom onset and the date and time of the enrollment survey.

⁎⁎ Statistical testing across arms performed using separate GEE models for each demographic characteristic and exchangeable correlation within household. The binary distribution and logit link function were used in all models with the exception of models for age, time since symptom onset, and hours between screening and enrollment survey, in which the Poisson distribution and log link function were used. P-values computed via Type 3 analyses.

### 3.2 Primary clinical outcomes

Two participants met the definition of LRTI at baseline. For the remaining high-risk participants, six (4.7%) progressed to LRTI (two control, 0 HCQ, four HCQ/AZ) (Table S4) and had sustained hypoxemia (<93%) for 1–9 days. Seven (5.4%) participants in the high-risk group had COVID-19 related hospitalizations with length of stay from 2–20 days (four control, one HCQ, two HCQ/AZ) (Table S5), of which two also reached the LRTI outcome. There were no episodes of LRTI and two (2.0%) COVID-19 related hospitalizations in the low-risk cohort (0 control, 1 HCQ, 1 HCQ/AZ). There were no deaths.

### 3.3 Primary virologic outcome

**Fig. 2:**
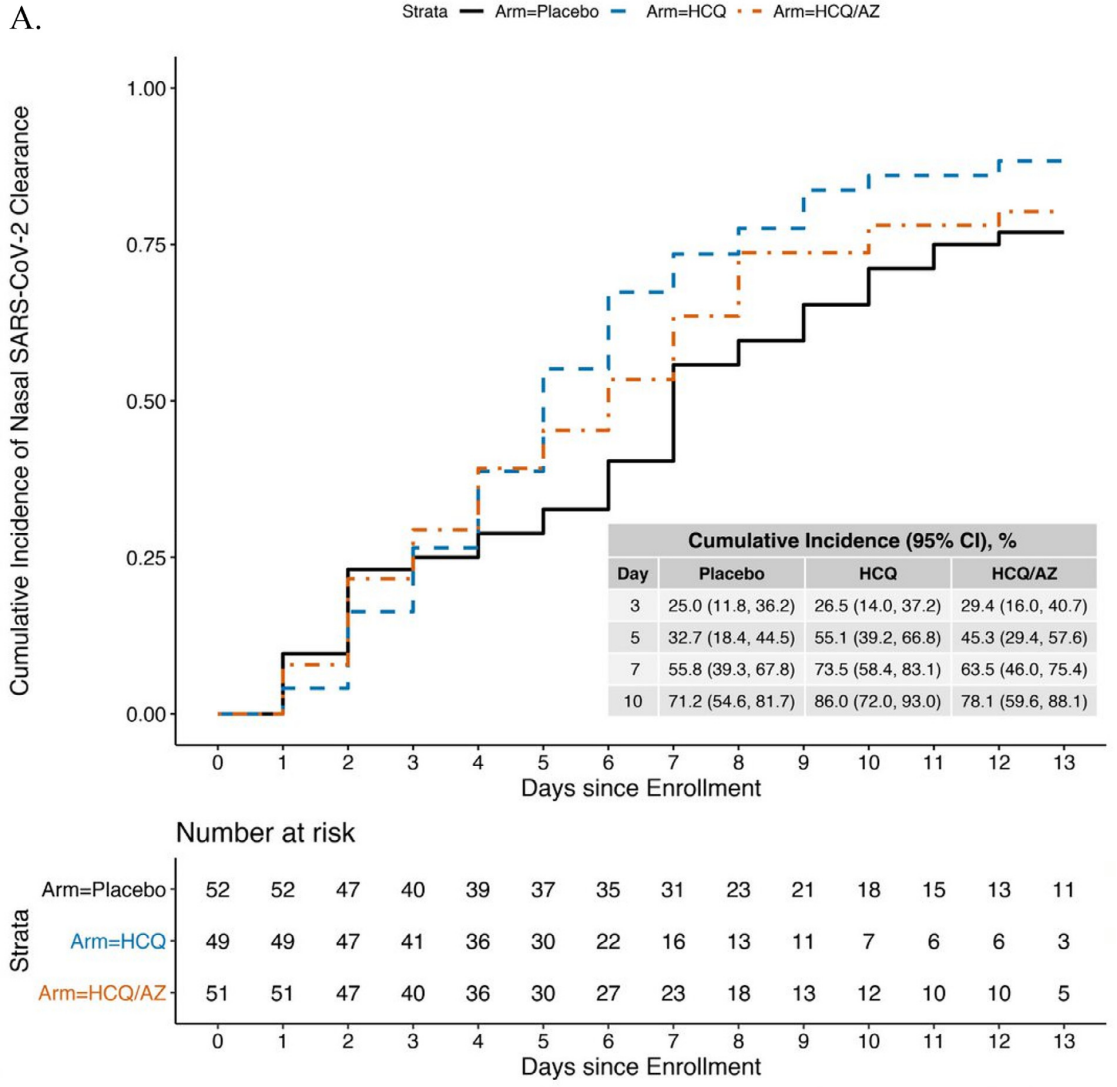

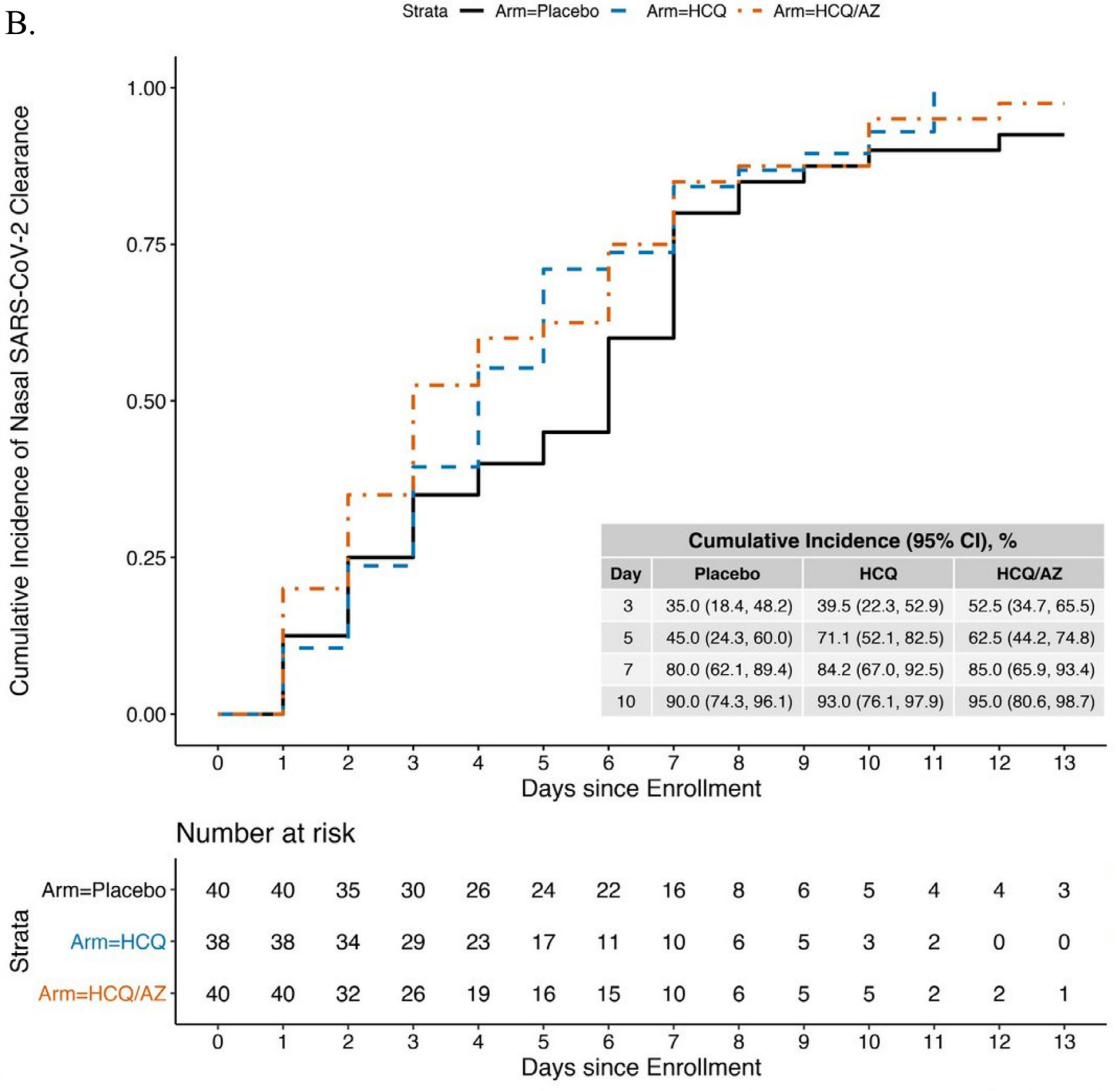
Kaplan-Meier curve showing time to viral clearance* over 14-days by study arm using a Ct cutoff of ≤40 (A) or ≤34 (B). *viral clearance defined as 2 consecutive swabs without SARS-CoV-2 detected.

Overall, 3109 of an expected 3696 (84.1%) nasal swabs were collected. Seventy-nine participants (34.2%) did not have SARS-CoV-2 detected or did not complete swabs on Days 1 or 2. The remaining 152 participants were randomized to HCQ (*n* = 49), HCQ/AZ (*n* = 51), and control (*n* = 52) and had similar characteristics by group at baseline (Table S6). RNaseP was performed on 586 swabs; only 6 (1.7%) had no RNaseP detected and were excluded. The remaining swabs had a median RNaseP Ct=25.9 (IQR=24.1–27.4) (data not shown). The median time to viral clearance was 7 days (95% CI=6–10 days) in the control group, 5 days (95% CI=4–6 days) in the HCQ group, and 6 days (95% CI=4–8) in the HCQ/AZ group (Fig. 2A). In the Cox model compared to the control group, clearance for the HCQ group was 62% faster (hazard ratio [HR] 1.62, 95% confidence interval [CI]=1.01–2.60, *p* = 0.047) and the HCQ/AZ group trended to faster clearance but did not reach statistical significance (HCQ/AZ: HR=1.25, 95% CI=0.75–2.07, *p* = 0.39) (Table 2).

**Table 2:** Cox proportional hazards model for time to clearance of nasal SARS-CoV-2 and time to COVID-19 symptom resolution, stratified by site and time since symptom onset (>4 days).

	N	N events	HR	95% CI	p-value

Viral clearance, SARS-CoV-2 Cutoff Ct≤40*					
Placebo	52	40	–	–	–
HCQ	49	43	**1.62**	**1.01–2.60**	**0.047**
HCQ/AZ	51	40	1.25	0.75–2.07	0.39
Viral clearance, SARS-CoV-2 Cutoff Ct≤34*					
Placebo	40	37	–	–	–
HCQ	38	37	1.15	0.72–1.85	0.55
HCQ/AZ	40	39	1.31	0.79–2.17	0.29
**COVID-19 symptom resolution**⁎⁎					
Placebo	72	38	–	–	–
HCQ	60	30	1.02	0.63–1.64	0.95
HCQ/AZ	65	31	0.91	0.57–1.45	0.70

⁎ *A priori*, stratified by study site and time of symptom onset, adjusted for age and sex.

⁎⁎ *A priori* stratified by time of symptom onset and high/low risk cohort, adjusted for age and sex.

Two post-hoc sensitivity analyses were conducted to interrogate the finding that HCQ was associated with slightly faster virologic clearance and to determine whether this would be predicted to have an impact on viral transmission. When we added baseline viral load (Ct) to the model, we found that HCQ was no longer significantly associated with decreased time to viral clearance (HR=1.50, 95% CI=0.89–2.52, *p* = 0.126), and the HCQ/AZ results were similar to the unadjusted model (HR=1.41, 95% CI=0.86–2.32, *p* = 0.174). A second sensitivity analysis, using a lower cycle time threshold Ct≤34, which is a more relevant threshold for viral transmission, included 118 participants (40 control, 38 HCQ, 40 HCQ/AZ) (Fig. 2B). Neither HCQ (HR=1.15, (95% CI=0.72–1.85, *p* = 0.55), nor HCQ/AZ was associated with faster time to clearance (HR=1.31, 95% CI=0.79–2.17, *p* = 0.29) (Table 2) in this model.

### 3.4 Time to symptom resolution

**Fig. 3:**
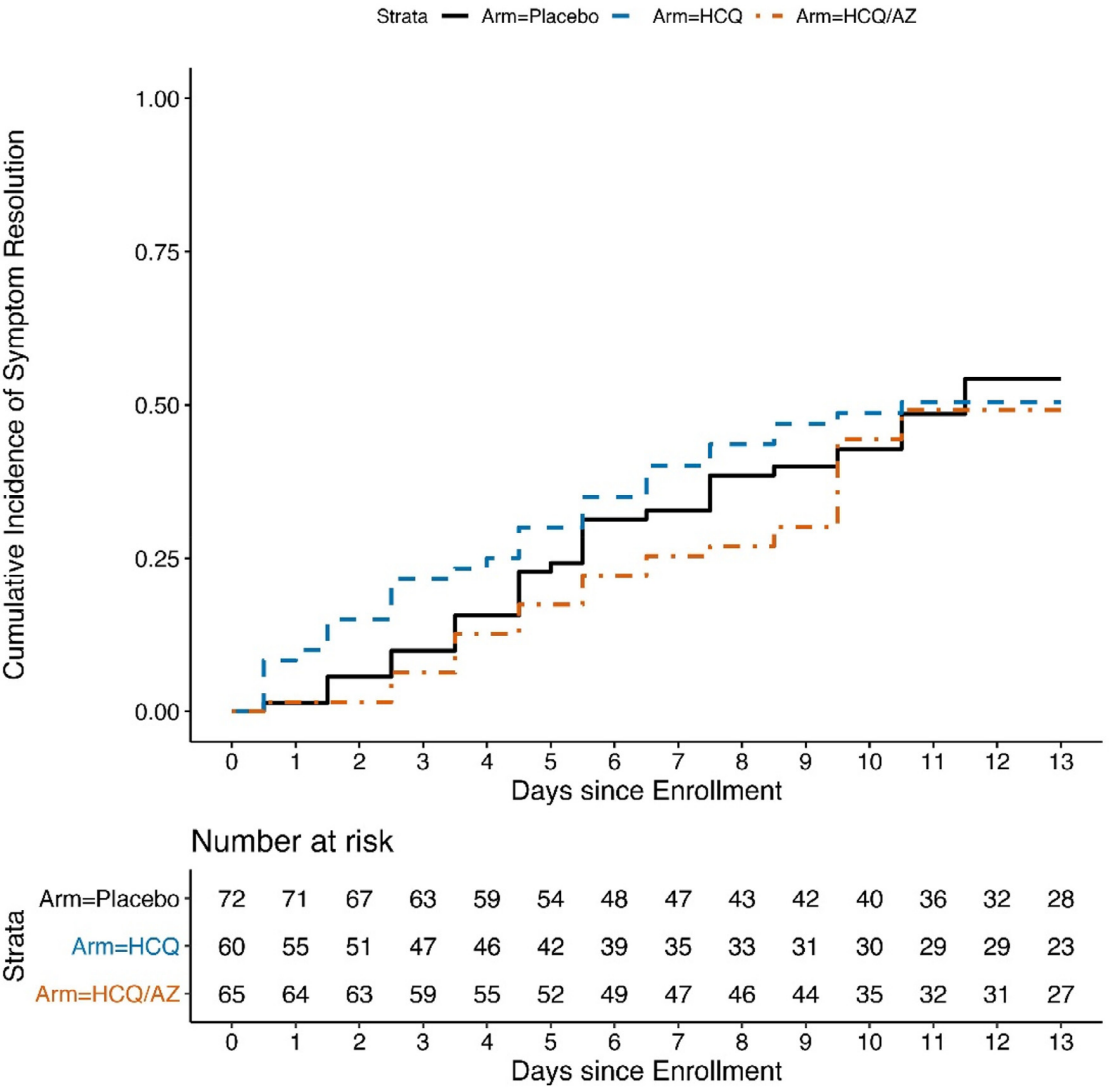
Time to resolution of COVID-19 symptoms by Day 14, by study arm.

The analysis included 197 participants (85.3%) who met the COVID-19 definition at screening (Table S7). The median time to symptom resolution was 11.5 days in the control group, 10.5 days in the HCQ group and the median time was not reached in the HCQ/AZ group (50.8% had resolved by Day 14). The upper limits of the 95% CIs were not evaluable. In the Cox model we found that neither HCQ (HR=1.02, 95% CI=0.63–1.64, *p* = 0.95) nor HCQ/AZ (HR=0.91, 95% CI=0.57–1.45, *p* = 0.70) were associated with faster resolution of COVID-19 symptoms (Table 2) Figure 3. Participants had similar symptoms ratings by randomized group by system over time (Figure S1A-G).

### 3.5 Vital signs monitoring

Among both high- and low-risk participants, the median O2 sat was 97% at baseline, did not vary throughout the 14-day follow up period and was similar by arm (Figure S1A). Similarly, the median baseline temperature (97.7–98.1), pulse (81–82 beats per minute), and respiratory rate (16–17 breaths per minute) did not differ by risk group or study arm over time (Figure S2-D).

### 3.6 Safety analyses

In the HCQ group there were three grade 1 or 2 AEs that were considered related to the study medication; in the HCQ/AZ group there were nineteen grade 1 or 2 related AEs (most (13/19, 68%) were GI related), and in the control group there were five grade 1 or 2 related AEs (Table 3). The prevalence and intensity of GI symptoms assessed by FluPro was similar in all randomized groups (Figure S1G). Overall, 11 (4.8%) participants had related AEs resulting in study medication discontinuation; 4 (5.6%) in the HCQ group, 5 (6.5%) in the HCQ/AZ group, and 2 (2.4%) in control group. The symptoms leading to discontinuation included nausea and/or vomiting (*n* = 3 HCQ/AZ, *n* = 1 HCQ, *n* = 2 control), headache (*n* = 1, HCQ), lower GI distress (*n* = 1, HCQ/AZ) and weakness (*n* = 1, HCQ/AZ), and hives/rash (*n* = 2 HCQ/AZ). Two participants in the HCQ arm discontinued study medication due to QTc prolongation>500 ms; both participants were asymptomatic, and no additional participants had QTc>500 ms during the trial. The median QTc was 408.5 at baseline and did not vary throughout the study, regardless of randomized group (Figure S3). In the HCQ group, there were 19 AEs for QTc change of >60 ms, and 7 in the HCQ/AZ group and 2 in the control group; there were no persistent QTc>60 ms on repeat EKG and all participants resumed study medication (Table 3).

**Table 3:** Adverse events during the study period.

	**Placebo**	**HCQ**	**HCQ /AZ**	**Total**

**Related AEs (non-EKG)**				
Severity Grade				
Grade 1/mild	3	1	8	12
Grade 2/moderate	2	2	11	15
Grade 3/severe	0	0	0	0
Grade 4/potentially -life threatening	0	0	0	0
Grade 5/death	0	0	0	0
Total	5	3	19	27
AEs due to EKG abnormalities*				
QTc>60 ms change from baseline*	2	19	7	28
QTc>500ms	–	2⁎⁎	–	2

⁎ No changes in QTc>60 ms from baseline were confirmed on repeat EKG. EKG abnormalities are not mutually exclusive: both QTc>500 ms events were also >60 ms change from baseline; however, only 1 RAE was reported in these cases, with >500 ms prioritized over >60 change from baseline.

⁎⁎ Resulted in study drug discontinuation.

## 4 Discussion

This 3-arm randomized, placebo-equivalent, remotely-conducted trial showed that HCQ or HCQ/AZ did not expedite resolution of COVID-19 symptoms compared to placebo-equivalent control among outpatients with COVID-19. We noted a modestly faster time to viral clearance with HCQ but not HCQ/AZ; however, this effect was not retained in sensitivity analyses, either adjusting for baseline viral load or excluding samples with low viral load that are unlikely to be significant for transmission. Given these findings, we caution against overinterpretation of the virologic data. We designed the trial to explore whether HCQ and HCQ/AZ decreased the frequency of disease progression to clinically significant endpoints – LRTI, hospitalization, and death. Although we recruited a population at substantial risk for developing severe COVID-19, few participants progressed, resulting in trial discontinuation for operational futility.

Although we were not able to analyze whether HCQ and HCQ/AZ decreased the risk of COVID-19 disease progression due to rare events, our findings are consistent with two published randomized trials of HCQ treatment of COVID-19 in outpatients; both trials showed that HCQ did not decrease the duration of COVID-19 symptoms [12,13]. A trial by Skipper et al. was conducted remotely in the US and similarly had few hospitalization endpoints and shifted the primary outcome to symptom resolution; there were no virologic outcomes in this study [12]. An open-label trial conducted in Spain found that HCQ was not associated with faster resolution of symptoms and the quantity of virus detected at three time points did not differ by randomization group [13]. Both studies recruited younger health care workers without risk factors for severe COVID-19. A third trial randomized, placebo-controlled trial by Omrani et al. showed HCQ with or without AZ was not associated with a difference in proportion of participants with viral clearance at day 6, among healthy young men recruited within one day of symptom onset [14]. The current trial extends these findings in a population enriched with high-risk participants with laboratory-confirmed infection and provides daily viral shedding and granular detail on COVID-19 symptoms, along with careful safety monitoring.

This is the second rigorously conducted randomized trial of HCQ/AZ among outpatients with SARS-CoV-2 infection. Our results indicate that HCQ/AZ does not hasten viral clearance as was hypothesized based on observational studies [8,9], similar to findings by Omrani et al. [14]. Perhaps more importantly, we did not find that HCQ/AZ decreases time to resolution of COVID-19 symptoms, a finding which is consistent with data from randomized controlled trials in hospitalized Brazilian patients with moderate or severe disease [24,25]. These data confirm that this combination has no benefit in outpatients with COVID-19. Early observational data from inpatients receiving HCQ/AZ suggested a high frequency of QTc prolongation [26]. We were able to rapidly operationalize a QTc monitoring system with near real-time clinically actionable feedback. In our study population, QTc prolongation was rare (0.9% participants with QTc>500 ms). More broadly, HCQ and HCQ/AZ were well tolerated and safe, although the combination of HCQ/AZ was associated with GI side effects.

We showed that rigorous clinical trials for treatment of COVID-19, with intensive virologic, vital sign, and cardiac monitoring, can be successfully conducted remotely with high retention and adherence to study protocol. The consistency of vital signs data suggests that remote collection is feasible and reliable. Remote clinical trials may provide many potential benefits for future studies including but not limited to: the ability to investigate infectious agents without risk of exposure, the massive expansion of enrollment catchment areas, and potential reduction in loss to follow-up. We hypothesize that the remote platform minimized some traditional barriers for recruitment, allowing for a diverse population of participants [27].

A limitation of the study design was that kit shipment increased the time from screening to study medication initiation. This may have affected measurement of the virologic outcome since a large proportion (34.2%) had already met the definition of viral clearance at enrollment. In addition, we used ascorbic acid as a placebo-equivalent rather than using a true placebo. While high doses of ascorbic acid are being studied for treatment of severe COVID-19 in the ICU setting, the low dose used in this trial is not expected to impact infection outcomes. Similarly, there are no in vitro data to suggest that the dose of folic acid used in this study would affect SARS-CoV-2 replication or viral clearance. However, we cannot rule out these doses of ascorbic acid and folic acid affected the clinical course of SARS-CoV-2 infection or viral replication. We also had several primary endpoints, but did not adjust for multiplicity, due to the concerns that endpoints were likely to be highly correlated. However, we cannot rule out the possibility that we spuriously found a statistically significant result.

Given that most COVID-19 infections occur in the outpatient setting, it is essential to continue to search for interventions that could alter the course of early disease and decrease risk of community transmission. Although we assumed that progression to severe COVID-19 would be a robust endpoint based on initial observations [28,29], we found that this was rare. Validated virologic or clinical risk factors for disease progression would greatly facilitate early treatment studies. Symptom resolution outcomes have been used in interventional trials of antivirals for influenza [30], and could be considered for outpatient COVID-19 trials.

As SARS-CoV-2 continues to disrupt life around the world, we must continue to search for therapies that reduce morbidity and transmission. Early treatment of SARS-CoV-2 infection remains an important goal for pandemic control [6]. Although this study showed that HCQ and HCQ/AZ are not effective in shortening the time to COVID-19 symptom resolution or decreasing viral shedding in a way that would interrupt transmission, important lessons from this trial can be applied to future trials of outpatient treatment for early SARS-CoV-2.

## Data sharing

A de-identified dataset will be available.

## Contributions

CJ, ERB, RVB, CC and JMB designed the trial. KBH, TQD, and ERB performed the data analyses and had full access to the full study data. CJ, HL, and SM oversaw the operations of the trial. CJ, ERB, and JMB wrote the first draft of the manuscript. All authors contributed equally to trial execution and interpretation of results. All authors approved the submitted manuscript.

## Declaration of Competing Interests

Declaration of interests: Grants from BMGF for conduct of the study (CJ, ERB, AS, SM, HL, AW, RVB, JMB), Declaration of interests outside of the submitted work: grants from BMGF (AB), grants from CDC (CJ), and NIH (CJ, ERB, HSK, SH, AB, AW), personal fees from Gilead (CJ, CC, AW, JMB) or grants from Gilead (ALG), personal fees from Merck (CC, AW, HYC) or grants from Merck (ALG), grants from Abbott (ALG) grants from Sanofi Pasteur (AW, HYC), grant from GSK (AW), travel from Innovative Molecules (AW), personal fees from Crozet, Aicuris, and X-vax (AW), personal fees from Medpace and AbbVie (CJ), personal fees from Gates Ventures (AB), personal fees for Pfizer, GSK, grant from Ellume (HYC), non-financial support Cepheid (HYC), JMB became an employee at Gilead outside and subsequent to the work.PAN and MJA have a potential financial relationship with AliveCor related to QT assessment using the device, however the investigators would receive no financial benefit for use of the technology for patients at Mayo Clinic or for its use in the current study. All other authors declare nothing.

## Appendix Supplementary materials

Image, application 1

## References

[1] Zhu N., Zhang D., Wang W. (2020). A novel coronavirus from patients with pneumonia in China, 2019. N Engl J Med.

[2] Dong E., Du H., Gardner L. (2020). An interactive web-based dashboard to track COVID-19 in real time. Lancet Infect Dis.

[3] Beigel J.H., Tomashek K.M., Dodd L.E. (2020). Remdesivir for the treatment of Covid-19 — final report. N Engl J Med.

[4] The RECOVERY Collaborative Group (2020). Dexamethasone in hospitalized patients with Covid-19 — preliminary report. N Engl J Med.

[5] Wu Z., McGoogan J.M., Characteristics of and Important Lessons From the Coronavirus Disease 2019 (2020). (COVID-19) outbreak in China: summary of a report of 72 314 cases from the chinese center for disease control and prevention. JAMA.

[6] Kim P.S., Read S.W., Fauci A.S. (2020). Therapy for early COVID-19: a critical need. JAMA.

[7] Yao X., Ye F., Zhang M. (2020). In vitro antiviral activity and projection of optimized dosing design of hydroxychloroquine for the treatment of severe acute respiratory syndrome Coronavirus 2 (SARS-CoV-2). Clin Infect Dis.

[8] Gautret P., Lagier J.C., Parola P. (2020). Hydroxychloroquine and azithromycin as a treatment of COVID-19: results of an open-label non-randomized clinical trial. Int J Antimicrob Agents.

[9] Lagier J.C., Million M., Gautret P. (2020). Outcomes of 3,737 COVID-19 patients treated with hydroxychloroquine/azithromycin and other regimens in Marseille, France: a retrospective analysis. Travel Med Infect Dis.

[10] Jean S.S., Lee P.I., Hsueh P.R. (2020). Treatment options for COVID-19: the reality and challenges. J Microbiol Immunol Infect.

[11] Pastick K.A., Okafor E.C., Wang F. (2020). Review: hydroxychloroquine and chloroquine for treatment of SARS-CoV-2 (COVID-19). Open Forum Infect Dis.

[12] Skipper C.P., Pastick K.A., Engen N.W. (2020). Hydroxychloroquine in non-hospitalized adults with early COVID-19: a randomized trial. Ann Intern Med.

[13] Mitjà O., Corbacho-Monné M., Ubals M. (2020). Hydroxychloroquine for early treatment of adults with mild Covid-19: a randomized-controlled trial. Clin Infect Dis.

[14] Omrani A.S., Pathan S.A., Thomas S.A. (2020). Randomized double-blinded placebo-controlled trial of hydroxychloroquine with or without azithromycin for virologic cure of non-severe Covid-19. EClinicalMedicine.

[15] Chu H.Y., Englund J.A., Starita L.M. (2020). Early detection of Covid-19 through a citywide pandemic surveillance platform. N Engl J Med.

[16] Powers J.H.III, Bacci E.D., Leidy N.K. (2018). Performance of the influenza patient-reported outcome (FLU-PRO) diary in patients with influenza-like illness (ILI). PLoS ONE.

[17] Harris P.A., Taylor R., Minor B.L. (2019). The REDCap consortium: building an international community of software platform partners. J Biomed Inform.

[18] Harris P.A., Taylor R., Thielke R., Payne J., Gonzalez N., Conde J.G. (2009). Research electronic data capture (REDCap)–a metadata-driven methodology and workflow process for providing translational research informatics support. J Biomed Inform.

[19] Lieberman J.A., Pepper G., Naccache S.N., Huang M.L., Jerome K.R., Greninger A.L. (2020). Comparison of commercially available and laboratory-developed assays for in vitro detection of SARS-CoV-2 in clinical laboratories. J Clin Microbiol.

[20] Centers for Diseae Control and Prevention. Coronavirus Disease (2019). (COVID-19) 2020 Interim Case Definiton, Approved April 5, 2020. 2020.

[21] Therneau T.M., Grambsch P.M. (2020). Modeling survival data: extending the cox model.

[22] La Scola B., Le Bideau M., Andreani J. (2020). Viral RNA load as determined by cell culture as a management tool for discharge of SARS-CoV-2 patients from infectious disease wards. Eur J Clin Microbiol Infect Dis.

[23] Gottlieb R.L., Nirula A., Chen P. (2021). Effect of Bamlanivimab as monotherapy or in combination with Etesevimab on viral load in patients with mild to moderate COVID-19. JAMA.

[24] Cavalcanti A.B., Zampieri F.G., Rosa R.G. (2020). Hydroxychloroquine with or without azithromycin in mild-to-moderate Covid-19. N Engl J Med.

[25] Furtado R.H.M., Berwanger O., Fonseca H.A. (2020). Azithromycin in addition to standard of care versus standard of care alone in the treatment of patients admitted to the hospital with severe COVID-19 in Brazil (COALITION II): a randomised clinical trial. Lancet.

[26] Chorin E., Dai M., Shulman E. (2020 Jun). The QT interval in patients with COVID-19 treated with hydroxychloroquine and azithromycin. Nat Med.

[27] Chastain D.B., Henao-Martinez A.F., Young H.N (2020). Racial disproportionality in COVID clinical trials. Reply. N Engl J Med.

[28] CDC Covid-19 Response Team. Severe Outcomes Among Patients with Coronavirus Disease 2019 (2020). (COVID-19) - United States, February 12-March 16, 2020. MMWR Morb Mortal Wkly Rep.

[29] Wortham J.M., Lee J.T., Althomsons S. (2020). Characteristics of persons who died with COVID-19 - United States, February 12-May 18, 2020. MMWR Morb Mortal Wkly Rep.

[30] Hayden F.G., Sugaya N., Hirotsu N. (2018). Baloxavir marboxil for uncomplicated influenza in adults and adolescents. N Engl J Med.

