# Supplementary material for: Hydroxychloroquine with or without azithromycin for treatment of early SARS-CoV-2 infection among high-risk outpatient adults: A randomized clinical trial
Source: eClinicalMedicine. 2021 Feb 27;33:100773. doi: 10.1016/j.eclinm.2021.100773 (PMC7912360; doi:10.1016/j.eclinm.2021.100773)
Supplement: Supplementary file 1 [file mmc1.docx]

**Hydroxychloroquine with or without azithromycin for treatment of early SARS-CoV-2 infection among high-risk outpatient adults: a randomized clinical trial**

Supplemental Figures and Tables.

Table of Contents

Supplemental Figures

Figure 1A-1G: Symptoms of COVID-19 over time by group………………………………….1

Figure 2A-2D: Vital signs over time by group………………………………………………….5

Figure 3: Median QTc over time by group……………………………………………………..7

Supplemental Tables

Table 1. Demographics in the high-risk cohort………………………………………………..8

Table 2. Demographics in the low-risk cohort………………………………………..............9

Table 3. Risk factors for high-risk cohort……………………………………………………..10

Table 4. Summary of persons who developed LRTI during the study……………………..11

Table 5. Summary of COVID-19 related hospitalizations…………………………………..11

Table 6. Demographics in the viral shedding cohort………………………………………...12

Table 7. Demographics in the disease resolution cohort…………………………………...14

Study sites and investigators……………………………………………………………………………............16

Supplemental Figure 1A. Overall FLU-Pro symptoms over time, by randomized group


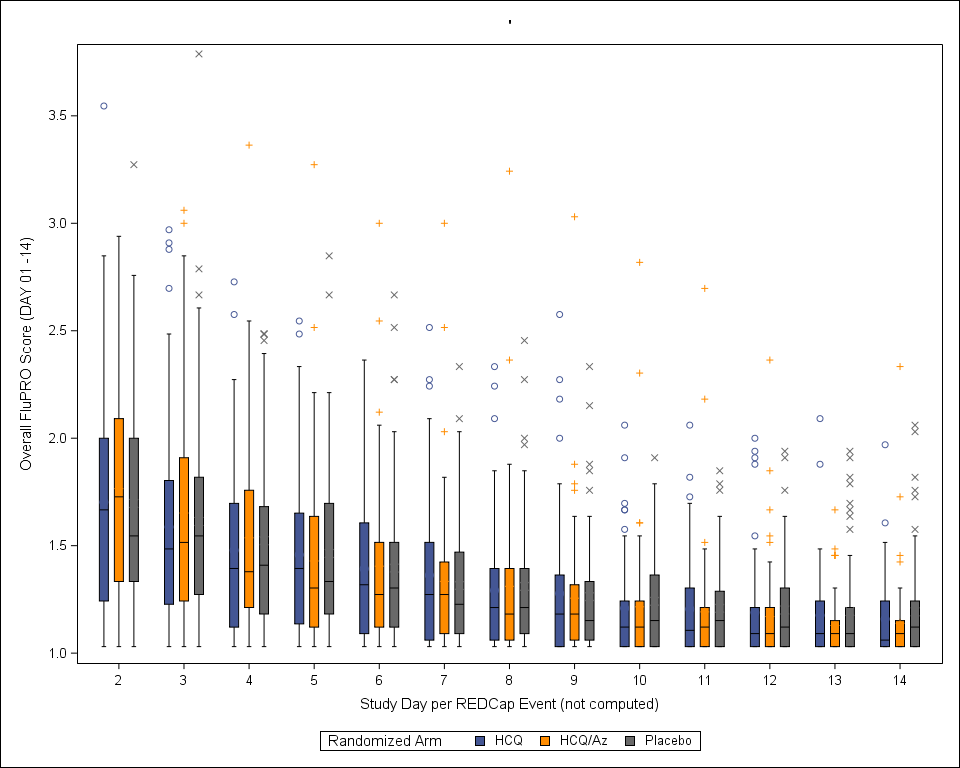


Supplemental Figure 1B. Systemic symptoms over time, by randomized group


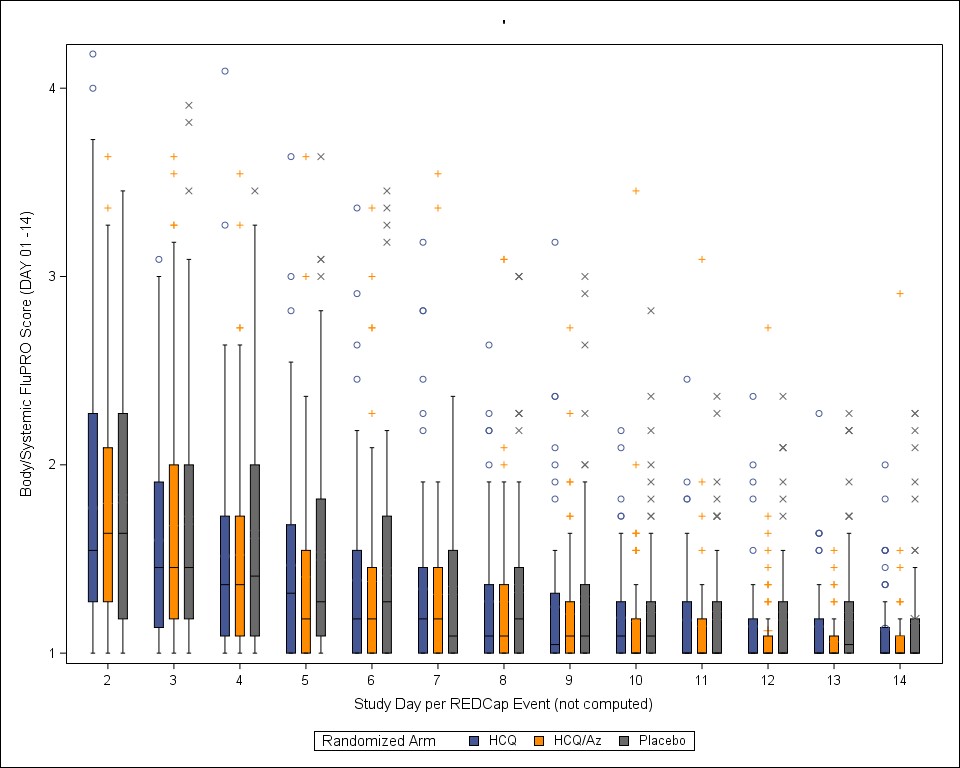


Supplemental Figure 1C. Chest symptoms over time, by randomized group


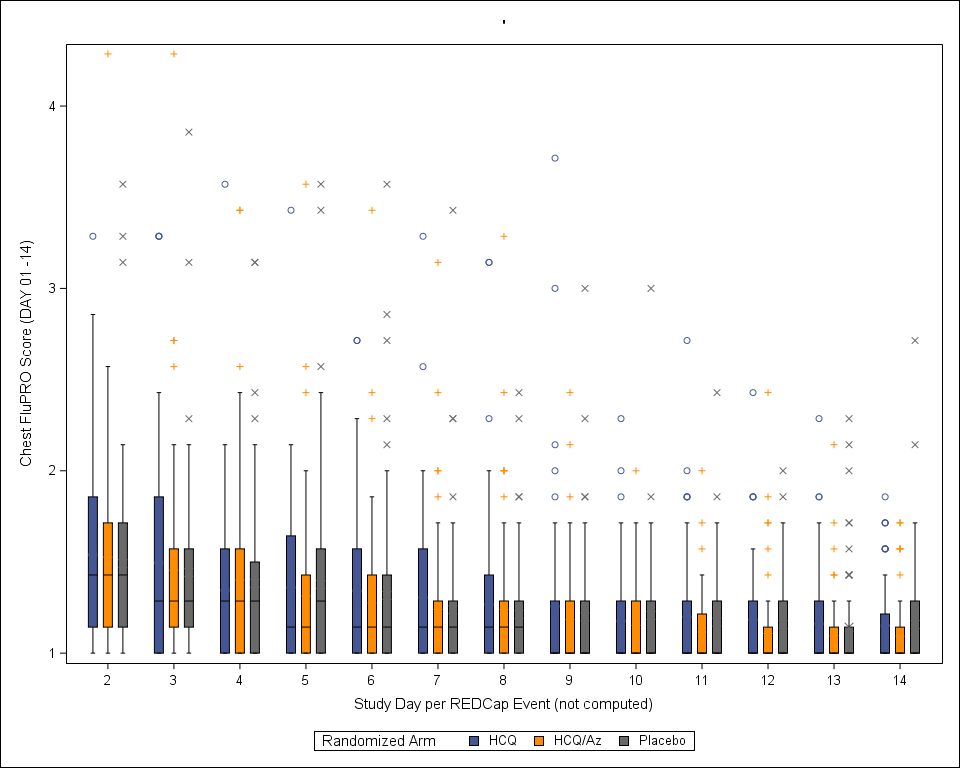


Supplemental Figure 1D. Nasal symptoms over time, by randomized group


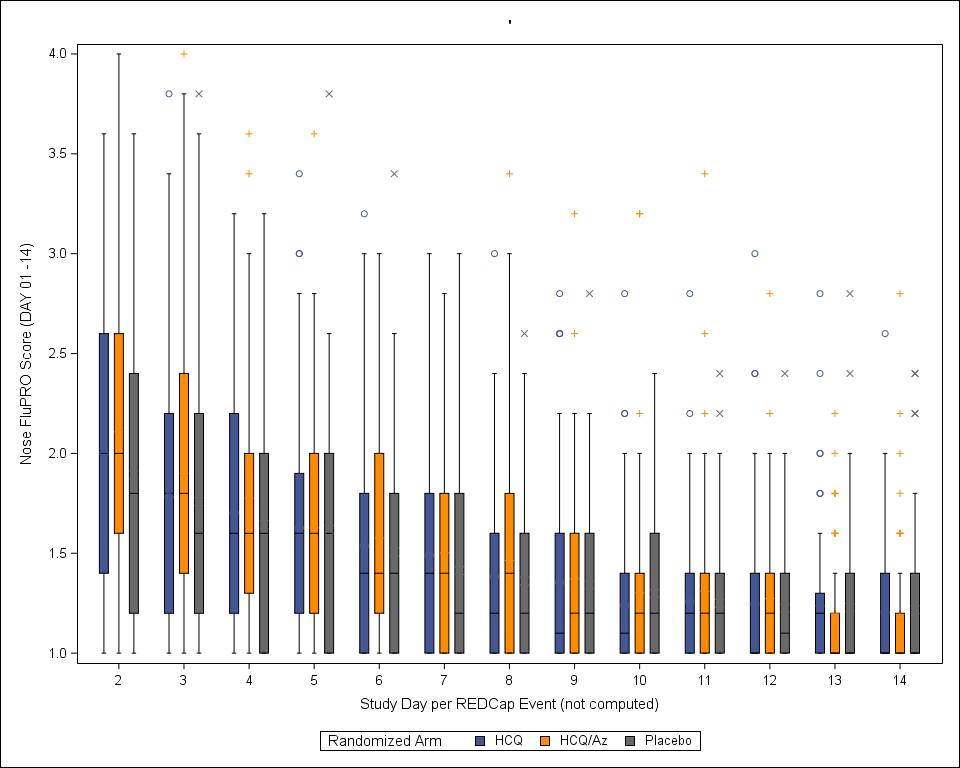


Supplemental Figure 1E. Ocular symptoms over time, by randomized group


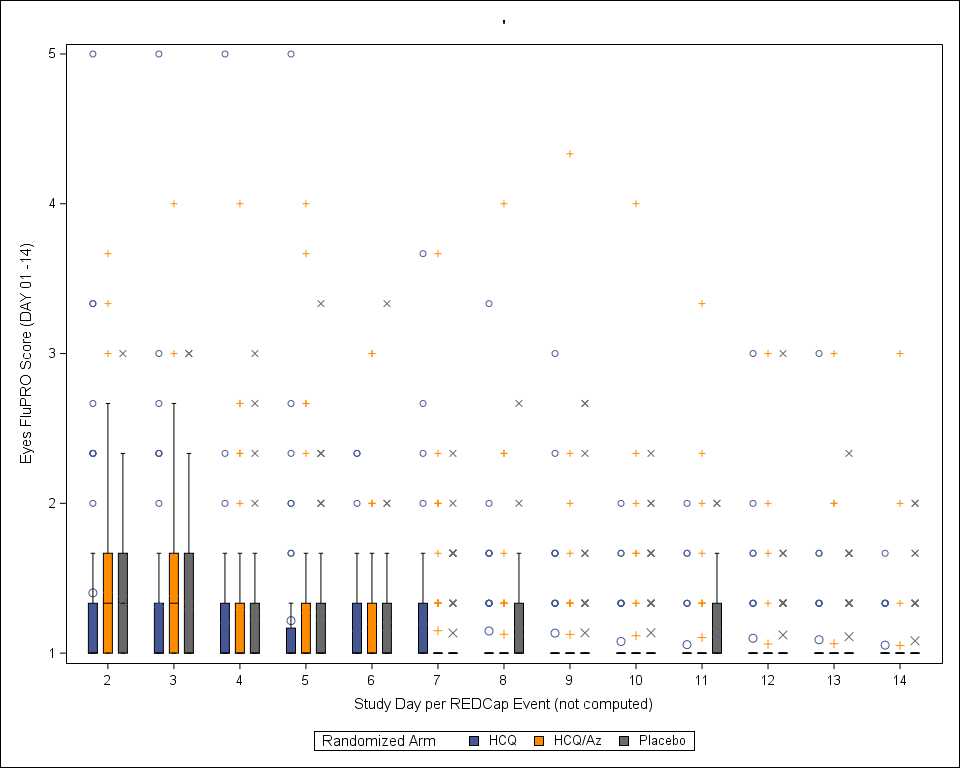


Supplemental Figure 1F. Throat symptoms over time, by randomized group


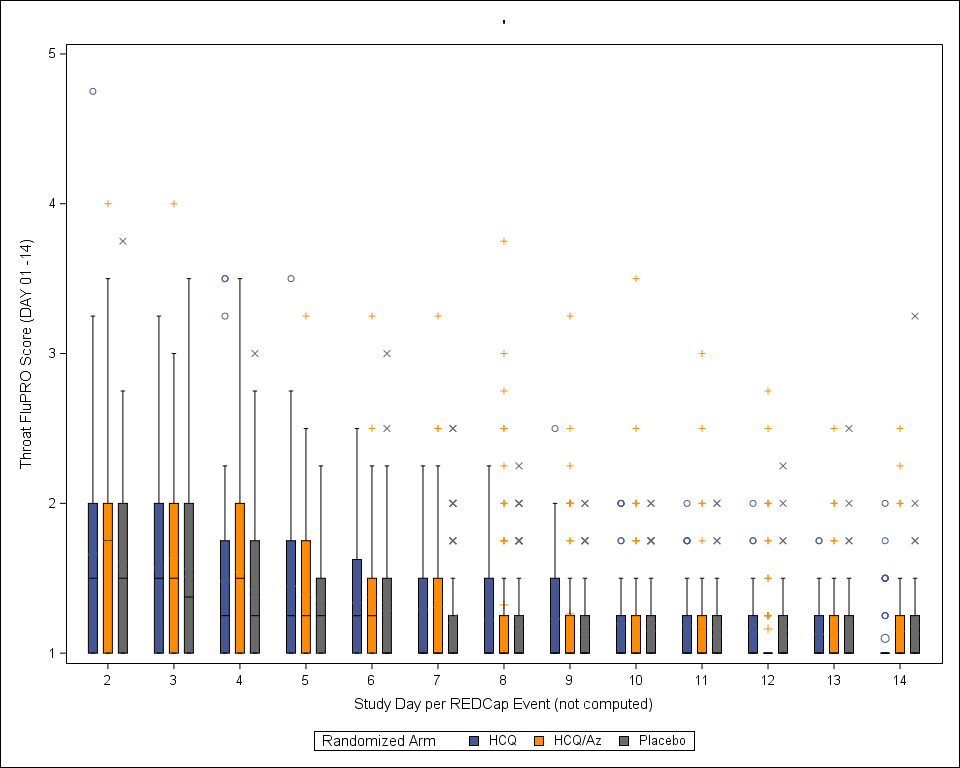


Supplemental Figure 1G. Gastrointestinal symptoms over time, by randomized group


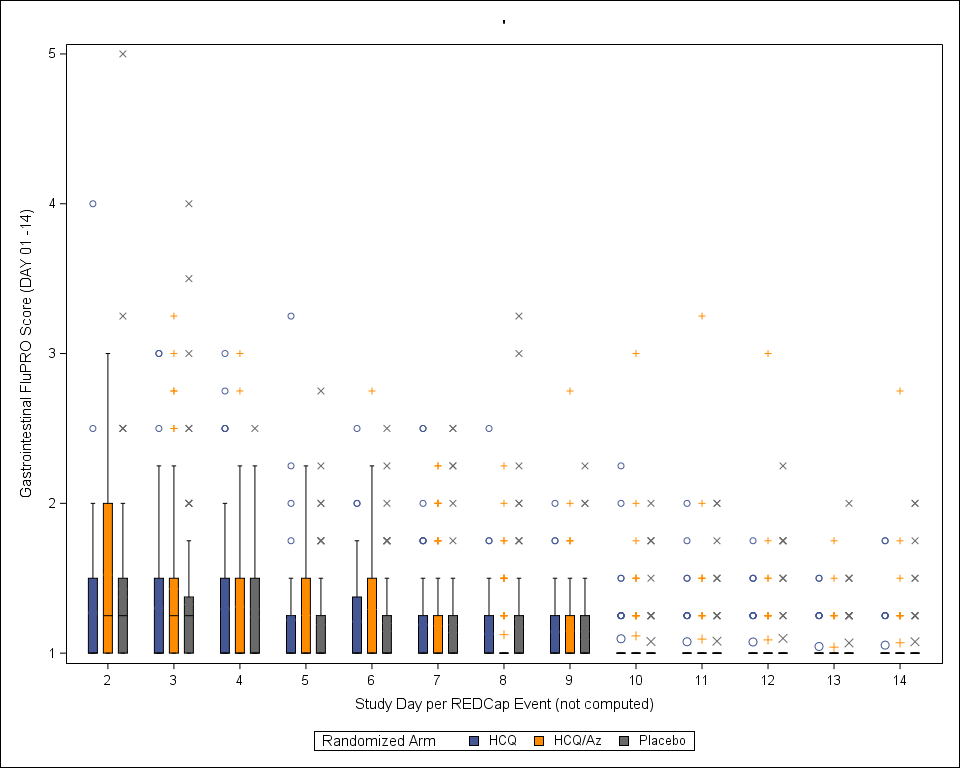


Supplemental Figure 2A. SpO2 over time, by randomized group


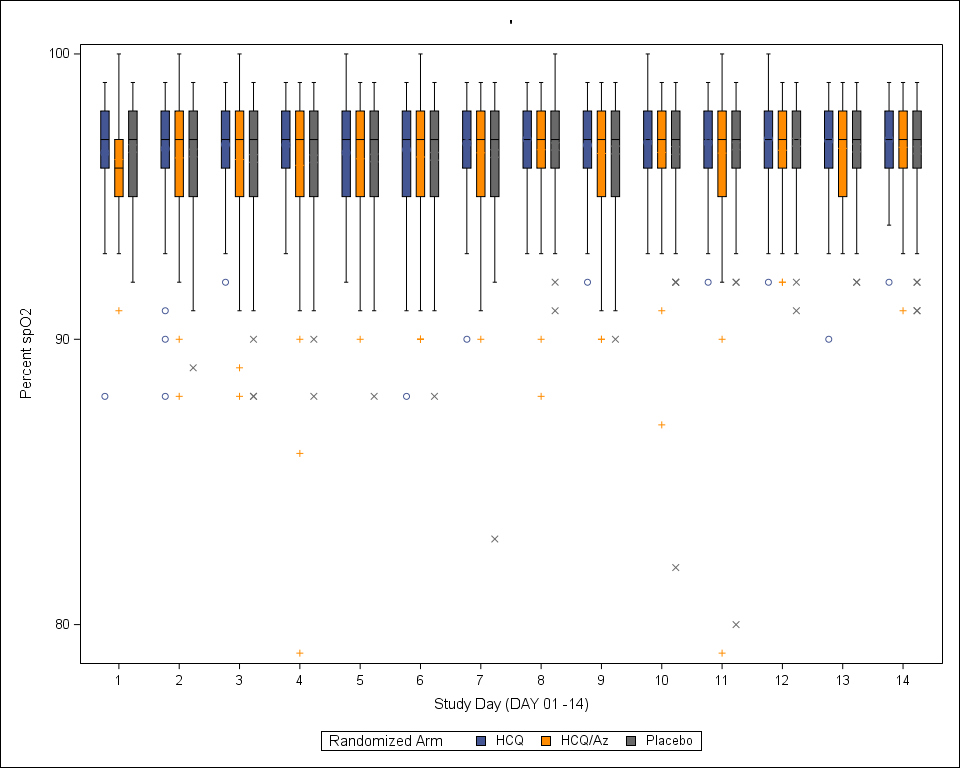


Supplemental Figure 2B. Pulse over time, by randomized group


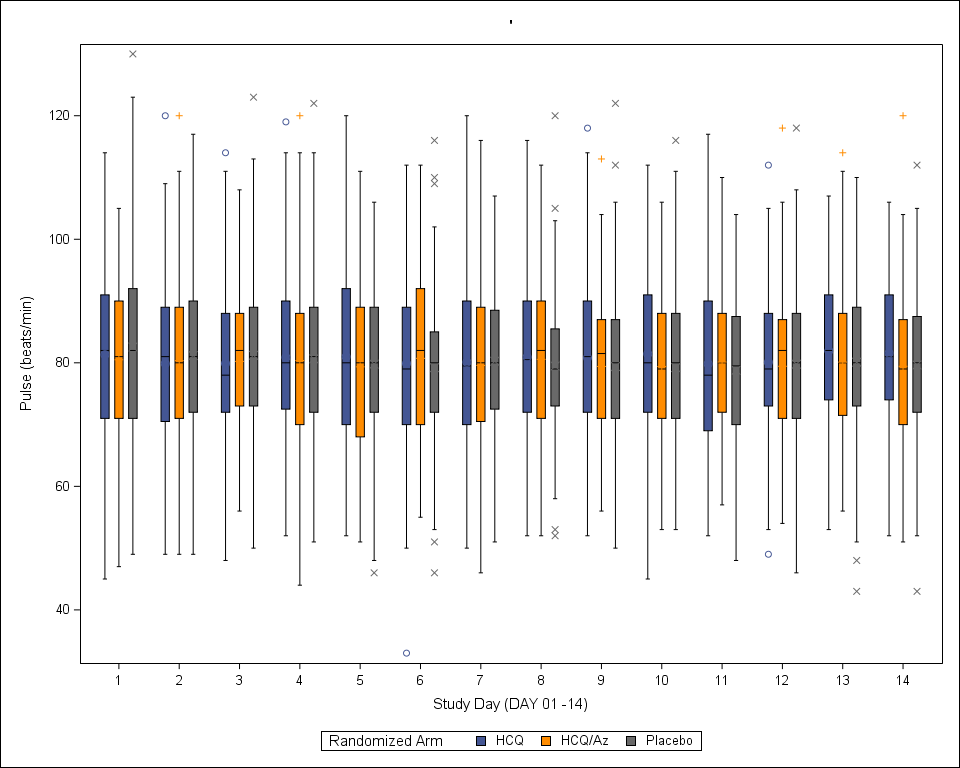


Supplemental Figure 2C. Respiratory rate over time, by randomized group


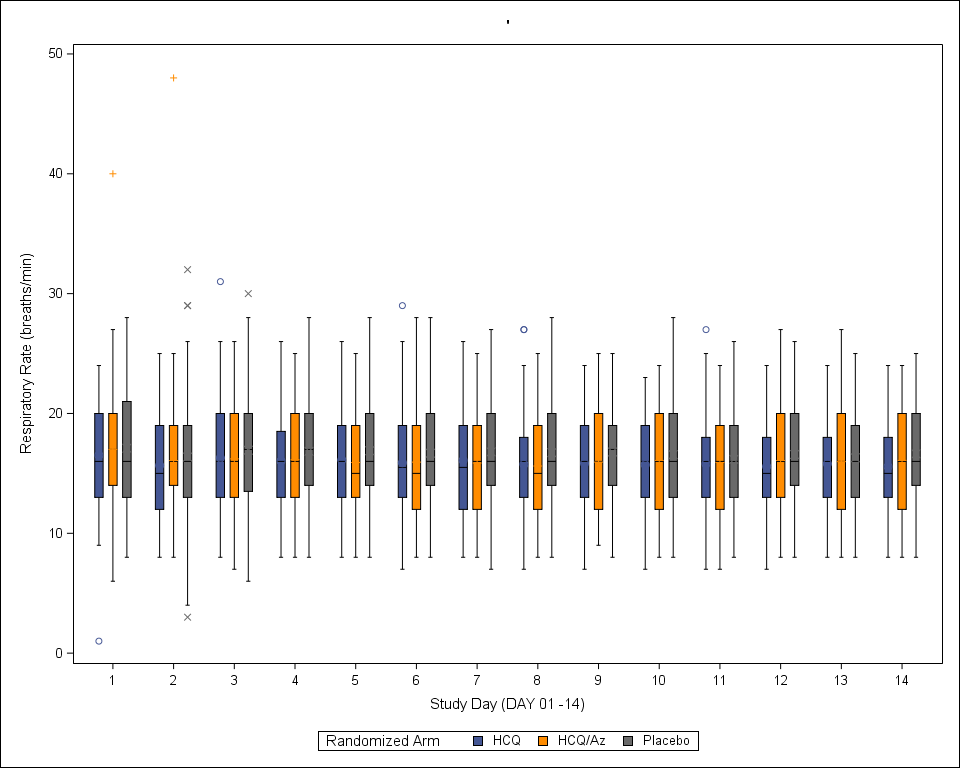


Supplemental Figure 2D. Temperature over time, by randomized group


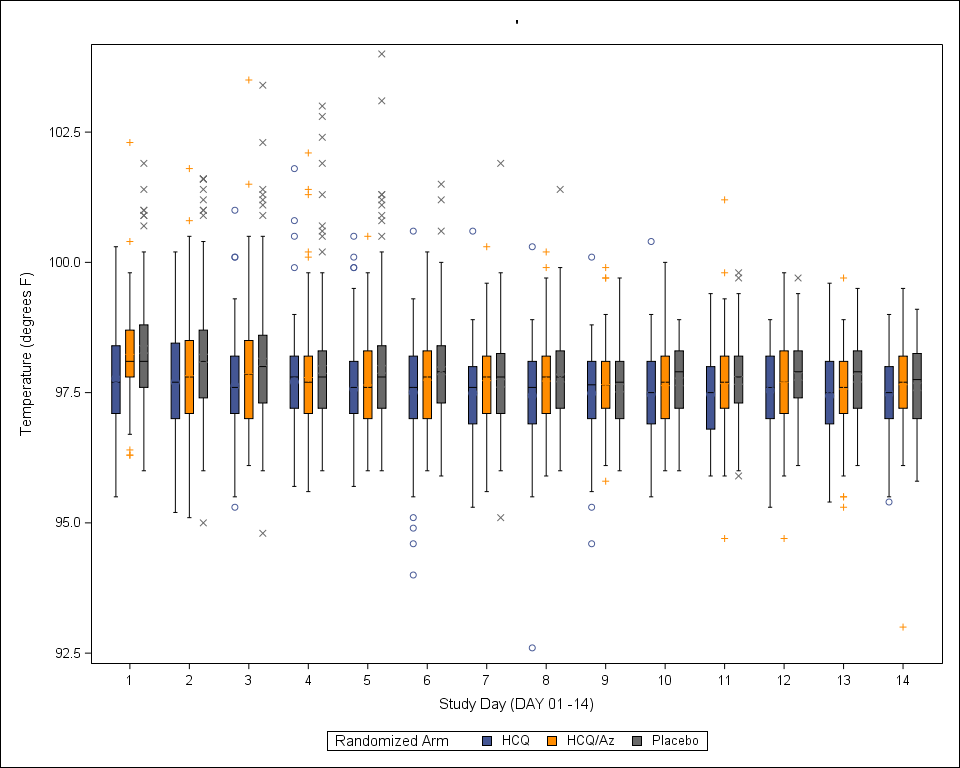


Supplemental Figure 3. QTc over time, by randomized group


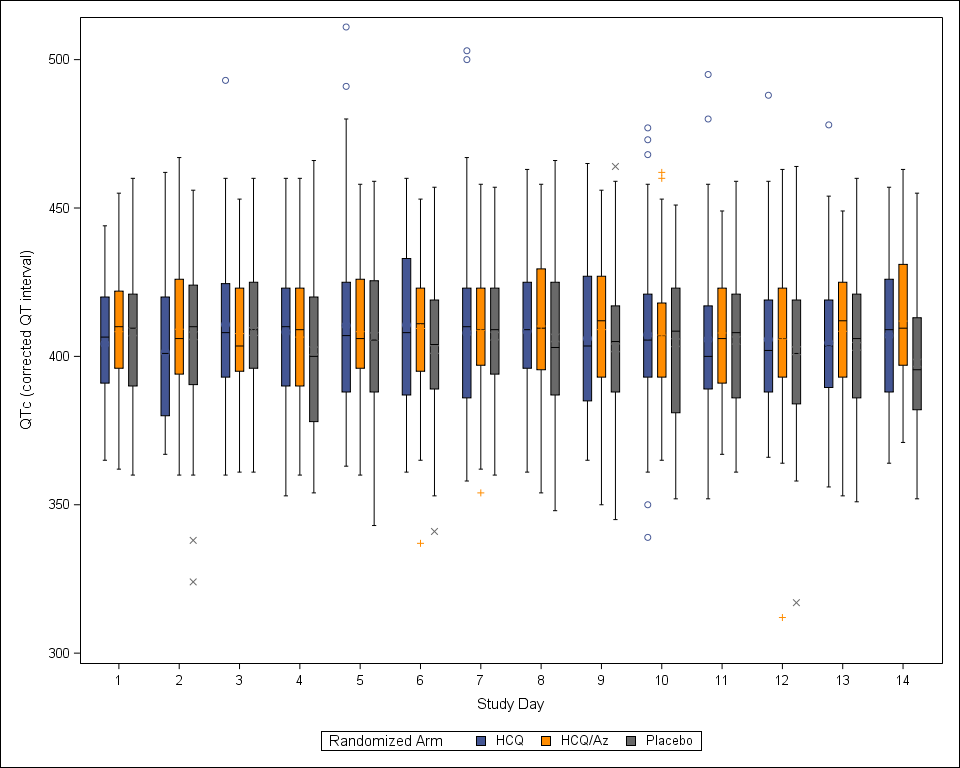


Supplemental Tables

Table 1. Demographics in high-risk cohort

|  |  |  |  |  |  |  |  |  |  |  |
| --- | --- | --- | --- | --- | --- | --- | --- | --- | --- | --- |
|  | | | **Randomized Arm** | | | | | **p-value** | |  |
|  | | | **Ascorbic acid+ folic acid** | **HCQ + folic acid** | **HCQ + azithromycin** | **All** | |  |  |  |
| **Characteristic** | | **Category** |  | | | | | | |  |
| Enrolled (n) | | Total | 48 | 37 | 44 | 129 | |  |  |  |
| Age (years) | | 18-29 | 7 (14.6%) | 4 (10.8%) | 10 (22.7%) | 21 (16.3%) | |  |  |  |
|  | | 30-39 | 14 (29.2%) | 18 (48.6%) | 9 (20.5%) | 41 (31.8%) | |  |  |  |
|  | | 40-49 | 11 (22.9%) | 6 (16.2%) | 8 (18.2%) | 25 (19.4%) | |  |  |  |
|  | | 50-59 | 8 (16.7%) | 3 (8.1%) | 8 (18.2%) | 19 (14.7%) | |  |  |  |
|  | | 60-69 | 7 (14.6%) | 5 (13.5%) | 8 (18.2%) | 20 (15.5%) | |  |  |  |
|  | | 70-80 | 1 (2.1%) | 1 (2.7%) | 1 (2.3%) | 3 (2.3%) | |  |  |  |
|  | | Median | 42 | 39 | 42 | 40 | |  | 0.8497 |  |
|  | | Min, max | (20, 70) | (19, 78) | (22, 71) | (19, 78) | |  |  |  |
| Sex | | Born Female | 26 (54.2%) | 23 (62.2%) | 25 (56.8%) | 74 (57.4%) | |  | 0.8336 |  |
|  | | Born Male | 22 (45.8%) | 14 (37.8%) | 19 (43.2%) | 55 (42.6%) | |  |  |  |
| Race | | American Indian or Alaska Native | 12 (25.0%) | 8 (21.6%) | 10 (22.7%) | 30 (23.3%) | |  |  |  |
|  | | Asian | 2 (4.2%) | 1 (2.7%) | 3 (6.8%) | 6 (4.7%) | |  |  |  |
|  | | Native Hawaiian or other Pacific Islander | 2 (4.2%) | 0 (0.0%) | 0 (0.0%) | 2 (1.6%) | |  |  |  |
|  | | Black or African American | 3 (6.3%) | 6 (16.2%) | 6 (13.6%) | 15 (11.6%) | |  |  |  |
|  | | White | 22 (45.8%) | 17 (45.9%) | 21 (47.7%) | 60 (46.5%) | |  |  |  |
|  | | Other | 6 (12.5%) | 5 (13.5%) | 3 (6.8%) | 14 (10.9%) | |  |  |  |
|  | | Prefer not to say | 1 (2.1%) | 0 (0.0%) | 1 (2.3%) | 2 (1.6%) | |  |  |  |
| Hispanic or Latina/Latino/Latinx | | No | 34 (70.8%) | 24 (64.9%) | 33 (75.0%) | 91 (70.5%) | |  | 0.4911 |  |
|  | | Yes | 13 (27.1%) | 13 (35.1%) | 11 (25.0%) | 37 (28.7%) | |  |  |  |
|  | | Prefer not to say | 1 (2.1%) | 0 (0.0%) | 0 (0.0%) | 1 (0.8%) | |  |  |  |
| Preferred Language | | English | 44 (91.7%) | 37 (100.0%) | 39 (88.6%) | 120 (93.0%) | |  |  |  |
|  | | Spanish | 4 (8.3%) | 0 (0.0%) | 5 (11.4%) | 9 (7.0%) | |  |  |  |
| BMI (kg/m2) | | <30 | 13 (27.1%) | 9 (24.3%) | 9 (20.5%) | 31 (24.0%) | |  | 0.7330 |  |
|  | | >=30 | 35 (72.9%) | 28 (75.7%) | 35 (79.5%) | 98 (76.0%) | |  |  |  |
| Symptomatic COVID-19 at screening* | | No | 3 (6.3%) | 1 (2.7%) | 2 (4.5%) | 6 (4.7%) | |  | 0.7118 |  |
|  | | Yes | 45 (93.8%) | 36 (97.3%) | 42 (95.5%) | 123 (95.3%) | |  |  |  |
| Time since symptom onset (days)* | | Median | 5.5 | 6.2 | 6.3 | 5.9 | |  | 0.8321 |  |
|  | | IQR | (3.9, 7.3) | (5.2, 7.8) | (4.0, 8.5) | (4.1, 7.9) | |  |  |  |
| Completed the enrollment survey (mITT) | | No | 3 (6.3%) | 4 (10.8%) | 2 (4.5%) | 9 (7.0%) | |  | 0.5841 |  |
|  | | Yes | 45 (93.8%) | 33 (89.2%) | 42 (95.5%) | 120 (93.0%) | |  |  |  |
| Hours between screening visit and enrollment survey completion | | <24 | 24 (61.5%) | 15 (44.1%) | 26 (65.0%) | 65 (57.5%) | |  | 0.6297 |  |
|  | | 24 - <48 | 14 (35.9%) | 14 (41.2%) | 13 (32.5%) | 41 (36.3%) |  | |  |  |
|  | | >=48 | 7 (17.9%) | 4 (11.8%) | 3 (7.5%) | 14 (12.4%) |  | |  |  |
| Households | | Total | 39 | 34 | 40 | 113 |  | |  |  |
| Number of participants per household | | 1 | 33 (84.6%) | 31 (91.2%) | 36 (90.0%) | 100 (88.5%) |  | |  |  |
|  | | 2 | 5 (12.8%) | 3 (8.8%) | 4 (10.0%) | 12 (10.6%) |  | |  |  |
|  | | 3+ | 1 (2.6%) | 0 (0.0%) | 0 (0.0%) | 1 (0.9%) |  | |  |  |
|  |  |  |  |  |  |  |  |  |  |  |
| *Criteria for symptomatic COVID-19 met at screening. Time since symptom onset computed as the difference in hours between date and time of (any) symptom onset and the date and time of the enrollment survey. | | | | | | | | | | |

**Statistical testing across arms performed using separate GEE models for each demographic characteristic and exchangeable correlation within household. The binary distribution and logit link function were used in all models with the exception of models for age, time since symptom onset, and hours between screening and enrollment survey, in which the Poisson distribution and log link function were used. P-values computed via Type 3 analyses.

Table 2. Demographics in low-risk cohort

|  | | | **Randomized Arm** | | | | **p-value** | |  |
| --- | --- | --- | --- | --- | --- | --- | --- | --- | --- |
|  | | | **Ascorbic acid+ folic acid** | **HCQ + folic acid** | **HCQ + azithromycin** | **All** |  |  |  |
| **Characteristic** | | **Category** |  | | | | | |  |
| Enrolled (n) | | Total | 35 | 34 | 33 | 102 |  |  |  |
| Age (years) | | 18-29 | 14 (40.0%) | 15 (44.1%) | 14 (42.4%) | 43 (42.2%) |  |  |  |
|  | | 30-39 | 12 (34.3%) | 10 (29.4%) | 12 (36.4%) | 34 (33.3%) |  |  |  |
|  | | 40-49 | 5 (14.3%) | 7 (20.6%) | 6 (18.2%) | 18 (17.6%) |  |  |  |
|  | | 50-59 | 4 (11.4%) | 2 (5.9%) | 1 (3.0%) | 7 (6.9%) |  |  |  |
|  | | 60-69 | 0 (0.0%) | 0 (0.0%) | 0 (0.0%) | 0 (0.0%) |  |  |  |
|  | | 70-80 | 0 (0.0%) | 0 (0.0%) | 0 (0.0%) | 0 (0.0%) |  |  |  |
|  | | Median | 33 | 31 | 33 | 32 |  | 0.6888 |  |
|  | | Min, max | (18, 59) | (19, 57) | (18, 52) | (18, 59) |  |  |  |
| Sex | | Born Female | 19 (54.3%) | 16 (47.1%) | 22 (66.7%) | 57 (55.9%) |  | 0.2652 |  |
|  | | Born Male | 16 (45.7%) | 18 (52.9%) | 11 (33.3%) | 45 (44.1%) |  |  |  |
| Race | | American Indian or Alaska Native | 0 (0.0%) | 3 (8.8%) | 6 (18.2%) | 9 (8.8%) |  |  |  |
|  | | Asian | 2 (5.7%) | 2 (5.9%) | 1 (3.0%) | 5 (4.9%) |  |  |  |
|  | | Native Hawaiian or other Pacific Islander | 0 (0.0%) | 0 (0.0%) | 1 (3.0%) | 1 (1.0%) |  |  |  |
|  | | Black or African American | 4 (11.4%) | 4 (11.8%) | 3 (9.1%) | 11 (10.8%) |  |  |  |
|  | | White | 19 (54.3%) | 20 (58.8%) | 18 (54.5%) | 57 (55.9%) |  |  |  |
|  | | Other | 9 (25.7%) | 5 (14.7%) | 4 (12.1%) | 18 (17.6%) |  |  |  |
|  | | Prefer not to say | 1 (2.9%) | 0 (0.0%) | 0 (0.0%) | 1 (1.0%) |  |  |  |
| Hispanic or Latina/Latino/Latinx | | No | 18 (51.4%) | 24 (70.6%) | 26 (78.8%) | 68 (66.7%) |  | 0.1322 |  |
|  | | Yes | 17 (48.6%) | 10 (29.4%) | 7 (21.2%) | 34 (33.3%) |  |  |  |
|  | | Prefer not to say | 0 (0.0%) | 0 (0.0%) | 0 (0.0%) | 0 (0.0%) |  |  |  |
| Preferred Language | | English | 28 (80.0%) | 31 (91.2%) | 31 (93.9%) | 90 (88.2%) |  | 0.1063 |  |
|  | | Spanish | 7 (20.0%) | 3 (8.8%) | 2 (6.1%) | 12 (11.8%) |  |  |  |
| BMI (kg/m2) | | <30 | 35 (100.0%) | 34 (100.0%) | 33 (100.0%) | 102 (100.0%) |  |  |  |
|  | | >=30 | 0 (0.0%) | 0 (0.0%) | 0 (0.0%) | 0 (0.0%) |  |  |  |
| Symptomatic COVID-19 at screening* | | No | 3 (8.6%) | 4 (11.8%) | 6 (18.2%) | 13 (12.7%) |  | 0.6124 |  |
|  | | Yes | 32 (91.4%) | 30 (88.2%) | 27 (81.8%) | 89 (87.3%) |  |  |  |
| Time since symptom onset (days)* | | Median | 6.8 | 5.2 | 5.2 | 5.6 |  | 0.0989 |  |
|  | | IQR | (4.8, 9.1) | (3.3, 7.5) | (3.7, 8.3) | (3.8, 8.3) |  |  |  |
| Completed the enrollment survey (mITT) | | No | 0 (0.0%) | 2 (5.9%) | 1 (3.0%) | 3 (2.9%) |  |  |  |
|  | | Yes | 35 (100.0%) | 32 (94.1%) | 32 (97.0%) | 99 (97.1%) |  |  |  |
| Hours between screening visit and enrollment survey completion | | <24 | 21 (67.7%) | 18 (56.3%) | 18 (62.1%) | 57 (62.0%) |  | 0.7728 |  |
|  | | 24 - <48 | 9 (29.0%) | 13 (40.6%) | 11 (37.9%) | 33 (35.9%) |  |  |  |
|  | | >=48 | 5 (16.1%) | 1 (3.1%) | 3 (10.3%) | 9 (9.8%) |  |  |  |
| Households | | Total | 31 | 32 | 29 | 92 |  |  |  |
| Number of participants per household | | 1 | 27 (87.1%) | 30 (93.8%) | 25 (86.2%) | 82 (89.1%) |  |  |  |
|  | | 2 | 4 (12.9%) | 2 (6.3%) | 4 (13.8%) | 10 (10.9%) |  |  |  |
|  | | 3+ | 0 (0.0%) | 0 (0.0%) | 0 (0.0%) | 0 (0.0%) |  |  |  |
|  |  |  |  |  |  |  |  |  |  |
| *Criteria for symptomatic COVID-19 met at screening. Time since symptom onset computed as the difference in hours between date and time of (any) symptom onset and the date and time of the enrollment survey. | | | | | | | | | |

**Statistical testing across arms performed using separate GEE models for each demographic characteristic and exchangeable correlation within household. The binary distribution and logit link function were used in all models with the exception of models for age, time since symptom onset, and hours between screening and enrollment survey, in which the Poisson distribution and log link function were used. P-values computed via Type 3 analyses.

Table 3. Risk factors for high-risk cohort

|  | **Arm** | | | | | **p-value*** | | |
| --- | --- | --- | --- | --- | --- | --- | --- | --- |
|  | **Ascorbic acid+ folic acid** | **HCQ +**  **folic acid** | **HCQ + AZ** | **All** | |  | |  |
| **Criteria** |  | | | | | | | |
| Participants Enrolled, n | 48 | 37 | 44 | 129 | |  |  | |
| Age ≥60 years, n(%) | 8 (16.7%) | 6 (16.2%) | 9 (20.5%) | 23 (17.8%) | |  | 0.8665 | |
|  |  |  |  |  | |  |  | |
| Presence of pulmonary disease, n(%) | 4 (8.3%) | 6 (16.2%) | 2 (4.5%) | 12 (9.3%) | |  | 0.2397 | |
| Moderate or severe persistent asthma | 1 (2.1%) | 0 (0.0%) | 1 (2.3%) | 2 (1.6%) | |  |  | |
| Chronic obstructive pulmonary disease/Emphysema | 1 (2.1%) | 2 (5.4%) | 1 (2.3%) | 4 (3.1%) | |  | 0.7271 | |
|  |  |  |  |  | |  |  | |
| Diabetes mellitus (Type 1) requiring oral medication or insulin for treatment | 1 (2.1%) | 0 (0.0%) | 1 (2.3%) | 2 (1.6%) | |  |  | |
| Diabetes mellitus (Type 2) required oral medication or insulin for treatment | 6 (12.5%) | 5 (13.5%) | 4 (9.1%) | 15 (11.6%) | |  | 0.7811 | |
|  |  |  |  |  | |  |  | |
| Hypertension, requiring at least 1 oral medication for treatment | 7 (14.6%) | 8 (21.6%) | 12 (27.3%) | 27 (20.9%) | |  | 0.3336 | |
|  |  |  |  |  | |  |  | |
| Immunocompromised status due to disease (for example, living with human immunodeficiency virus with a CD4 T-cell count of <200mm3) | 0 (0.0%) | 0 (0.0%) | 1 (2.3%) | 1 (0.8%) | |  |  | |
| Immunocompromised status due to medication | 1 (2.1%) | 1 (2.7%) | 0 (0.0%) | 2 (1.6%) | |  |  | |
|  |  |  |  |  | |  |  | |
| BMI ≥30 | 35 (72.9%) | 28 (75.7%) | 35 (79.5%) | 98 (76.0%) | |  | 0.7330 | |
|  |  |  |  |  | |  |  | |
| Number of risk factors per person |  |  |  |  | |  |  | |
| 1 | 37 (77.1%) | 25 (67.6%) | 28 (63.6%) | 90 (69.8%) | |  | 0.3945 | |
| 2 | 6 (12.5%) | 6 (16.2%) | 11 (25.0%) | 23 (17.8%) | |  |  | |
| 3 or more | 5 (10.4%) | 6 (16.2%) | 5 (11.4%) | 16 (12.5%) | |  |  | |
| *P-values computed using separate GEE models for each high-risk criteria and exchangeable correlation within household. The binary distribution and logit link function were used and p-values computed via Type 3 analyses. | | | | |  |  | |  |
|  | | | | | | | | |

Table 4. Summary of persons who developed LRTI during the study

|  | | | | | | | | **O2<93%** | |  |
| --- | --- | --- | --- | --- | --- | --- | --- | --- | --- | --- |
| **Randomized Arm** | | **Age (y)** | **Sex at birth** | **Study Day of LRTI*** | **Days since symptom onset**** | **Study Days with O2 measurements completed** | **Study Days**  **With O2<93% (n)** | | **% days with O2<93%** | |
| Ascorbic acid+ folic acid | | 61 | Male | 1 | 3.1 | 16 | 13 | | 81.3% | |
|  | | 25 | Female | 5 | 6.3 | 16 | 1 | | 6.3% | |
|  | | 58 | Male | 3 | 23.0 | 14 | 7 | | 50.0% | |
| HCQ + folic acid | | 56 | Female | 1 | 13.8 | 16 | 2 | | 12.5% | |
| HCQ + azithromycin | | 35 | Female | 3 | 8.9 | 16 | 3 | | 18.8% | |
|  | | 50 | Male | 9 | 3.4 | 15 | 3 | | 20.0% | |
|  | | 65 | Male | 3 | 7.2 | 9 | 7 | | 77.8% | |
|  | | 68 | Female | 5 | 9.1 | 16 | 9 | | 56.3% | |
|  |  |  |  |  |  |  |  |  |  |  |
| * Participants with LRTI at study day 1 were excluded from analysis. | | | | | | | | | |  |
| **Computed as days from symptom start date to enrollment (Day 01) survey completion. | | | | | | | | | |  |

Table 5. Summary of COVID-19 related hospitalizations

| **Randomized Arm** | | **Risk Cohort** | **Age (y)** | **Sex at birth** | **Days since symptom onset*** | **Study Day of Hospitalization** | **Adverse event** | **Days Hospitalized (n)** | **LRTI prior to hospitalization** |  |
| --- | --- | --- | --- | --- | --- | --- | --- | --- | --- | --- |
| Ascorbic acid+ folic acid | | High Risk | 37 | Female | 2.2 | 1 | COVID Pneumonia | 5 | No |  |
|  | | High Risk | 58 | Male | 23.0 | 24 | Pneumonia | 20 | Yes |  |
|  | | High Risk | 46 | Male | 9.2 | 5 | chest pain, chest burning | 4 | No |  |
|  | | High Risk | 36 | Male | 5.5 | 3 | bilateral pneumonia | 11 | No |  |
| HCQ + folic acid | | High Risk | 40 | Male | 9.0 | 14 | pericarditis | 2 | No |  |
|  | | Low Risk | 43 | Female |  | 0 | Chest Pain | 3 | No |  |
| HCQ + azithromycin | | High Risk | 57 | Female | 6.2 | 8 | COVID-19 pneumonia | 6 | No |  |
|  | | High Risk | 65 | Male | 7.2 | 9 | Respiratory distress associated with COVID 19 | 7 | Yes |  |
|  | | Low Risk | 35 | Female | 2.5 | 4 | COVID-related hemorrhagic colitis | 2 | No |  |
|  |  |  |  |  |  |  |  |  |  |  |
| *Adjudicated by endpoints committee. | | | | | | | | | | |
| *Computed as days from symptom start date to enrollment (Day 01) survey completion. Missing for 1 participant who did not complete the  enrollment survey. | | | | | | | | | | |

Table 6. Demographics in Viral Shedding Cohort

|  | | | **Randomized Arm** | | | | **p-value**** | |  |
| --- | --- | --- | --- | --- | --- | --- | --- | --- | --- |
|  | | | **Ascorbic acid+ folic acid** | **HCQ + folic acid** | **HCQ + azithromycin** | **All** |  |  | |
| **Characteristic** | | **Category** |  | | | | | |  |
| Enrolled (n) | | Total | 52 | 49 | 51 | 152 |  |  | |
| Age (years) | | 18-29 | 13 (25.0%) | 16 (32.7%) | 15 (29.4%) | 44 (28.9%) |  |  | |
|  | | 30-39 | 14 (26.9%) | 20 (40.8%) | 13 (25.5%) | 47 (30.9%) |  |  | |
|  | | 40-49 | 11 (21.2%) | 7 (14.3%) | 11 (21.6%) | 29 (19.1%) |  |  | |
|  | | 50-59 | 7 (13.5%) | 4 (8.2%) | 6 (11.8%) | 17 (11.2%) |  |  | |
|  | | 60-69 | 6 (11.5%) | 2 (4.1%) | 6 (11.8%) | 14 (9.2%) |  |  | |
|  | | 70-80 | 1 (1.9%) | 0 (0.0%) | 0 (0.0%) | 1 (0.7%) |  |  | |
|  | | Median | 39 | 33 | 37 | 37 |  | 0.0729 | |
|  | | Min, max | (18, 70) | (19, 68) | (18, 66) | (18, 70) |  |  | |
| Sex | | Born Female | 27 (51.9%) | 27 (55.1%) | 31 (60.8%) | 85 (55.9%) |  | 0.7351 | |
|  | | Born Male | 25 (48.1%) | 22 (44.9%) | 20 (39.2%) | 67 (44.1%) |  |  | |
| Race | | American Indian or Alaska Native | 11 (21.2%) | 9 (18.4%) | 13 (25.5%) | 33 (21.7%) |  |  | |
|  | | Asian | 2 (3.8%) | 2 (4.1%) | 0 (0.0%) | 4 (2.6%) |  |  | |
|  | | Native Hawaiian or other Pacific Islander | 1 (1.9%) | 0 (0.0%) | 1 (2.0%) | 2 (1.3%) |  |  | |
|  | | Black or African American | 1 (1.9%) | 5 (10.2%) | 5 (9.8%) | 11 (7.2%) |  |  | |
|  | | White | 29 (55.8%) | 28 (57.1%) | 28 (54.9%) | 85 (55.9%) |  |  | |
|  | | Other | 6 (11.5%) | 5 (10.2%) | 4 (7.8%) | 15 (9.9%) |  |  | |
|  | | Prefer not to say | 2 (3.8%) | 0 (0.0%) | 0 (0.0%) | 2 (1.3%) |  |  | |
| Hispanic or Latina/Latino/Latinx | | No | 34 (65.4%) | 34 (69.4%) | 40 (78.4%) | 108 (71.1%) |  | 0.3801 | |
|  | | Yes | 17 (32.7%) | 15 (30.6%) | 11 (21.6%) | 43 (28.3%) |  |  | |
|  | | Prefer not to say | 1 (1.9%) | 0 (0.0%) | 0 (0.0%) | 1 (0.7%) |  |  | |
| Preferred Language | | English | 45 (86.5%) | 46 (93.9%) | 46 (90.2%) | 137 (90.1%) |  | 0.4029 | |
|  | | Spanish | 7 (13.5%) | 3 (6.1%) | 5 (9.8%) | 15 (9.9%) |  |  | |
| BMI (kg/m2) | | <30 | 31 (59.6%) | 29 (59.2%) | 30 (58.8%) | 90 (59.2%) |  | 0.8227 | |
|  | | ≥30 | 21 (40.4%) | 20 (40.8%) | 21 (41.2%) | 62 (40.8%) |  |  | |
| Symptomatic COVID-19 at screening* | | No | 2 (3.8%) | 3 (6.1%) | 4 (7.8%) | 9 (5.9%) |  | 0.9206 | |
|  | | Yes | 50 (96.2%) | 46 (93.9%) | 47 (92.2%) | 143 (94.1%) |  |  | |
| Time since symptom onset (days)* | | Median | 5.3 | 5.5 | 5.3 | 5.4 |  | 0.8297 | |
|  | | IQR | (3.4, 7.2) | (4.1, 7.2) | (3.9, 7.9) | (3.9, 7.3) |  |  | |
| Completed enrollment | | No | 0 (0.0%) | 0 (0.0%) | 0 (0.0%) | 0 (0.0%) |  |  | |
|  | | Yes | 52 (100.0%) | 49 (100.0%) | 51 (100.0%) | 152 (100.0%) |  |  | |
| Hours between screening visit and enrollment survey completion | | <24 | 32 (78.0%) | 23 (50.0%) | 33 (71.7%) | 88 (66.2%) |  | 0.3494 | |
|  | | 24 - <48 | 14 (34.1%) | 23 (50.0%) | 16 (34.8%) | 53 (39.8%) |  |  | |
|  | | ≥48 | 6 (14.6%) | 3 (6.5%) | 2 (4.3%) | 11 (8.3%) |  |  | |
| Households | | Total | 41 | 46 | 46 | 133 |  |  | |
| Number of participants per household | | 1 | 32 (78.0%) | 42 (91.3%) | 39 (84.8%) | 113 (85.0%) |  |  | |
|  | | 2 | 7 (17.1%) | 3 (6.5%) | 5 (10.9%) | 15 (11.3%) |  |  | |
|  | | 3+ | 1 (2.4%) | 0 (0.0%) | 0 (0.0%) | 1 (0.8%) |  |  | |
| Medication Adherence | |  | 890/988 (90.1%) | 816/931 (87.6%) | 815/969 (84.1%) | 2521/2888 (87.3%) |  | 0.5062 | |
| Swab Completion | |  | 751/832 (90.3%) | 723/784 (92.2%) | 737/816 (90.3%) | 2211/2432 (90.9%) |  |  | |
|  |  |  |  |  |  |  |  |  |  |
| *Criteria for symptomatic COVID-19 met at screening. Time since symptom onset computed as the difference in hours between date and time of (any) symptom onset and the date and time of the enrollment survey.  **Statistical testing across arms performed using separate GEE models for each demographic characteristic and exchangeable correlation within household. The binary distribution and logit link function were used in all models with the exception of models for age, time since symptom onset, and hours between screening and enrollment survey, in which the Poisson distribution and log link function were used. P-values computed via Type 3 analyses. | | | | | | | | |  |

Table 7. Demographics in Disease Resolution Cohort

|  | | | | | **Randomized Arm** | | | | **p-value*** | |  |  |
| --- | --- | --- | --- | --- | --- | --- | --- | --- | --- | --- | --- | --- |
|  | | | | | **Ascorbic acid+ folic acid** | **HCQ + folic acid** | **HCQ + azithromycin** | **All** |  |  |  |  |
| **Characteristic** | | | | **Category** |  | | | | | |  |  |
| Enrolled (n) | | | | Total | 72 | 60 | 65 | 197 |  |  |  |  |
| Age (years) | | | | 18-29 | 19 (26.4%) | 16 (26.7%) | 18 (27.7%) | 53 (26.9%) |  |  |  |  |
|  | | | | 30-39 | 25 (34.7%) | 26 (43.3%) | 17 (26.2%) | 68 (34.5%) |  |  |  |  |
|  | | | | 40-49 | 10 (13.9%) | 10 (16.7%) | 13 (20.0%) | 33 (16.8%) |  |  |  |  |
|  | | | | 50-59 | 11 (15.3%) | 5 (8.3%) | 9 (13.8%) | 25 (12.7%) |  |  |  |  |
|  | | | | 60-69 | 6 (8.3%) | 3 (5.0%) | 7 (10.8%) | 16 (8.1%) |  |  |  |  |
|  | | | | 70-80 | 1 (1.4%) | 0 (0.0%) | 1 (1.5%) | 2 (1.0%) |  |  |  |  |
|  | | | | Median | 36 | 35 | 38 | 37 |  | 0.1900 |  |  |
|  | | | | Min, max | (18, 70) | (19, 68) | (18, 71) | (18, 71) |  |  |  |  |
| Sex | | | | Born Female | 39 (54.2%) | 34 (56.7%) | 41 (63.1%) | 114 (57.9%) |  | 0.7541 |  |  |
|  | | | | Born Male | 33 (45.8%) | 26 (43.3%) | 24 (36.9%) | 83 (42.1%) |  |  |  |  |
| Race | | | | American Indian or Alaska Native | 12 (16.7%) | 11 (18.3%) | 15 (23.1%) | 38 (19.3%) |  |  |  |  |
|  | | | | Asian | 4 (5.6%) | 3 (5.0%) | 2 (3.1%) | 9 (4.6%) |  |  |  |  |
|  | | | | Native Hawaiian or other Pacific Islander | 2 (2.8%) | 0 (0.0%) | 1 (1.5%) | 3 (1.5%) |  |  |  |  |
|  | | | | Black or African American | 4 (5.6%) | 7 (11.7%) | 7 (10.8%) | 18 (9.1%) |  |  |  |  |
|  | | | | White | 36 (50.0%) | 31 (51.7%) | 34 (52.3%) | 101 (51.3%) |  |  |  |  |
|  | | | | Other | 12 (16.7%) | 8 (13.3%) | 5 (7.7%) | 25 (12.7%) |  |  |  |  |
|  | | | | Prefer not to say | 2 (2.8%) | 0 (0.0%) | 1 (1.5%) | 3 (1.5%) |  |  |  |  |
| Hispanic or Latina/Latino/Latinx | | | | No | 47 (65.3%) | 41 (68.3%) | 51 (78.5%) | 139 (70.6%) |  | 0.2532 |  |  |
|  | | | | Yes | 24 (33.3%) | 19 (31.7%) | 14 (21.5%) | 57 (28.9%) |  |  |  |  |
|  | | | | Prefer not to say | 1 (1.4%) | 0 (0.0%) | 0 (0.0%) | 1 (0.5%) |  |  |  |  |
| Preferred Language | | | | English | 64 (88.9%) | 57 (95.0%) | 61 (93.8%) | 182 (92.4%) |  | 0.3655 |  |  |
|  | | | | Spanish | 8 (11.1%) | 3 (5.0%) | 4 (6.2%) | 15 (7.6%) |  |  |  |  |
| BMI (kg/m2) | | | | <30 | 44 (61.1%) | 34 (56.7%) | 35 (53.8%) | 113 (57.4%) |  | 0.5125 |  |  |
|  | | | | ≥30 | 28 (38.9%) | 26 (43.3%) | 30 (46.2%) | 84 (42.6%) |  |  |  |  |
| Symptomatic COVID-19 at screening* | | | | No | 0 (0.0%) | 0 (0.0%) | 0 (0.0%) | 0 (0.0%) |  | 0.9206 |  |  |
|  | | | | Yes | 72 (100.0%) | 60 (100.0%) | 65 (100.0%) | 197 (100.0%) |  |  |  |  |
| Time since symptom onset (days)* | | | | Median | 5.9 | 5.9 | 5.5 | 5.9 |  | 0.6383 |  |  |
|  | | | | IQR | (4.0, 8.2) | (4.0, 7.8) | (3.9, 8.2) | (3.9, 8.1) |  |  |  |  |
| Completed enrollment | | | | No | 0 (0.0%) | 0 (0.0%) | 0 (0.0%) | 0 (0.0%) |  |  |  |  |
|  | | | | Yes | 72 (100.0%) | 60 (100.0%) | 65 (100.0%) | 197 (100.0%) |  |  |  |  |
| Hours between screening visit and enrollment survey completion | | | | <24 | 40 (67.8%) | 31 (55.4%) | 37 (63.8%) | 108 (62.4%) |  | 0.5063 |  |  |
|  | | | | 24 - <48 | 22 (37.3%) | 24 (42.9%) | 23 (39.7%) | 69 (39.9%) |  |  |  |  |
|  | | | | ≥48 | 10 (16.9%) | 5 (8.9%) | 5 (8.6%) | 20 (11.6%) |  |  |  |  |
| Households | | | | Total | 59 | 56 | 58 | 173 |  |  |  |  |
| Number of participants per household | | | | 1 | 49 (83.1%) | 52 (92.9%) | 51 (87.9%) | 152 (87.9%) |  |  |  |  |
|  | | | | 2 | 9 (15.3%) | 4 (7.1%) | 7 (12.1%) | 20 (11.6%) |  |  |  |  |
|  | | | | 3+ | 1 (1.7%) | 0 (0.0%) | 0 (0.0%) | 1 (0.6%) |  |  |  |  |
| Medication adherence | | | |  | 1189/1368 (86.9%) | 989/1140 (86.8%) | 1037/1235 (84.0%) | 3215/3743  (85.9%) |  | 0.7754 |  |  |
| Swab Completion | | | |  | 1021/1152 (88.6%) | 880/960 (91.7%) | 938/1040 (90.2%) | 2339/3152 (90.1%) |  |  |  |  |
|  | | | |  |  |  |  |  |  |  |  |  |
|  |  | |  |  |  |  |  |  |  |  |  |  |
|  | | *Criteria for symptomatic COVID-19 met at screening. Time since symptom onset computed as the difference in hours between date and time of  (any) symptom onset and the date and time of the enrollment survey.  **Statistical testing across arms performed using separate GEE models for each demographic characteristic and exchangeable correlation within household. The binary distribution and logit link function were used in all models with the exception of models for age, time since symptom onset, and hours between screening and enrollment survey, in which the Poisson distribution and log link function were used. P-values computed via Type 3 analyses. | | | | | | | | | | |

COVID-19 Early Treatment Team

| **Boston Medical Center**  Luisa Arroyave, Agata Bereznicka, Jonathan Berz, Pablo Buitron, Michael Camuso, Leticia Cardoso, Ricardo Cruz, Julien Dedier, Husam Dennaoui, Anna Goldman, Lori Henault, Terrell Johnson, Sarah Koberna, Carlie Martinez, Erin Martinez, Crystal Ng, Michael Paasche-Orlow, Margot Rogers, Kathleen Salerno, Carl Streed, Ve Truong, Nisha Verma, Katherine Waite, Steven Zalewski  **Fred Hutchinson Cancer Research Center**  Elizabeth R. Brown, Tracy Q. Dong, Joshua Schiffer  **John H. Stroger, Jr. Hospital of Cook County**  Chris Balthazar, Kelly Bojan, Hamid Bouiri, Marisol Consignado, Kortez Davis, Sadhana Dharmapuri, Mireya Gonzalez, Sybil Hosek, Rachel Jackson, Meenakshi Malhotra, Antionette McFadden-Smith, Raymond McPherson, Ryan Muench, Ixchell Oritz-Estes, Temitope Oyedele, Dorothy Rego, Zoe Ellen Sanders, Alisa Seo-Lee, Karen Simpson,  **Mayo Clinic**  Michael J. Ackerman, Zachi I. Attia, Peter A. Noseworthy  **NYU Grossman School of Medicine**  Stefanie E. Bendik, Anna Bershteyn, Robert A. Pitts  **SUNY Upstate Medical University**  Peter Greco, Michelle Klick, Kristopher M. Paolino  **Tulane University**  Mueenah Anibaba, Evan Atkinson, Mary Beth Campbell, John Dwyer, Gerard Gomes, Jacob Hall, John Huntwork, Margaret Huntwork, Patricia Kissinger, Heather Larkin, Cedrick Ntambwe, Florice Numbi, Michelle Paloomares, Norine Schmidt, Hamada Rady, Maria Ribando, Daniel Triggs, Neha Upadhyay, Crystal Zheng |
| --- |

**University of Washington,** Department of Medicine, Division of Allergy and Infectious Diseases Medhavi Bole, Alyssa Braun, Helen Y. Chu, Mark Drummond, Kirsten Hauge, Madelaine Humphreys, Abir Hussein, Christine Johnston, Steve Kuntz, Anya Mathur, Lindsey McClellan, Jessica Moreno, Thepthara Pholsena, Matthew Seymour, Helen Stankiewicz-Karita, Jenell Stewart, Jina Taub, Zoe Thuesmunn, Ethan Valinetz, Dana Varon, Anna Wald, Brian Wood;

University of Washington, Institute of Translational Health Sciences, Maianna Dematteis & Katie Wicklander;

University of Washington, Department of Medicine, Division of Cardiology, Rebecca Letterer, Jeanne Poole, Arun R. Sridhar;

University of Washington, Department of Pharmacy, Jeff Purcell

University of Washington, Department of Medicine, International Training and Education Center for Health, Mary Kirk & Chloe D. Waters;

University of Washington ICRC, Department of Global Health: Jared M. Baeten, Ruanne V. Barnabas, Jennifer Baugh, Clare E. Brown, Connie Celum, Daphne Hamilton, Harald S. Haugen, Kate B. Heller, Rachel Johnson, Jack Knauer, Hannah Leingang, Caroline H. Liou, Susan Morrison, Justice Quame-Amaglo, Azaad, Randy Stalter, Jenell Stewart, Katherine Thomas, Vianey Vazquez, Grant E. Young, Yasaman Zia, Azaad Zimmermann

University of Washington, Department of Laboratory Medicine, Meei-Li Huang, Alexander L. Greninger, Keith R. Jerome, Mark H. Wener

**Virginia Mason Memorial Hospital**

Deborah J. Brown, Nathaniel Davenport, Omar Gambito
